# HIV Clinical Pathway: A New Approach to Combine Guidelines and Sustainability of Anti-Retroviral Treatment in Italy

**DOI:** 10.1371/journal.pone.0168399

**Published:** 2016-12-28

**Authors:** Davide Croce, Adriano Lazzarin, Giuliano Rizzardini, Nicola Gianotti, Francesca Scolari, Emanuela Foglia, Elisabetta Garagiola, Elena Ricci, Teresa Bini, Tiziana Quirino, Paolo Viganò, Tiziana Re, Antonella D’Arminio Monforte, Paolo Bonfanti

**Affiliations:** 1 Centre for Research in Health Economics, Social and Health Care Management, LIUC-Università Cattaneo, Varese, Italy; 2 School of Public Health, Faculty of Health Sciences, University of the Witwatersrand, Johannesburg, South Africa; 3 Clinic of Infectious Diseases, San Raffaele Scientific Institute, Milan, Italy; 4 Department of Infectious Diseases, Fatebebefratelli Sacco Hospital, Milan, Italy; 5 School of Clinical Medicine, Faculty of Health Science, University of the Witwatersrand, Johannesburg, South Africa; 6 Unit of Infectious Diseases, Santi Paolo e Carlo Hospital, Milan, Italy; 7 Department of Infectious Diseases, Valle Olona Hospital, Busto Arsizio, Italy; 8 Department of Infectious Diseases, Ovest Milanese Hospital, Legnano, Italy; 9 Department of Infectious Diseases, Lecco Hospital, Lecco, Italy; Universita degli Studi di Roma Tor Vergata, ITALY

## Abstract

The present article describes the case study of a “real world” HIV practice within the debate concerning the strategic role of Clinical Governance (CG) tools in the management of a National Healthcare System’s sustainability. The study aimed at assessing the impact of a Clinical Pathway (CP) implementation, required by the Regional Healthcare Service, in terms of effectiveness (virological and immunological conditions) and efficiency (economic resources absorption), from the budget holder perspective. Data derived from a multi-centre cohort of patients treated in 6 Hospitals that provided care to approximately 42% of the total HIV+ patients, in Lombardy Region, Italy. Two phases were compared: Pre-CP (2009–2010) *vs*. Post-CP implementation (2011–2012). All HIV infected adults, observed in the participating hospitals during the study periods, were enrolled and stratified into the 3 categories defined by the Regional CP: first-line, switch for toxicity/other, and switch for failure. The study population was composed of 1,284 patients (Pre-CP phase) and 1,135 patients (Post-CP phase). The results showed that the same level of virological and immunological effectiveness was guaranteed to HIV+ patients: 81.2% of Pre-CP phase population and 83.2% of Post-CP phase population had undetectable HIV-RNA (defined as <50 copies/mL) at 12-month follow up. CD4^+^ cell counts increased by 28 ± 4 cells/mm^3^ in Pre-CP Phase and 39 ± 5 cells/mm^3^ in Post-CP Phase. From an economic point of view, the CP implementation led to a substantial advantage: the mean total costs related to the management of the HIV disease (ART, hospital admission and laboratory tests) decreased (-8.60%) in the Post-CP phase (p-value < 0.0001). Results confirmed that the CP provided appropriateness and quality of care, with a cost reduction for the budget holder.

## Introduction

The introduction of Highly Active Anti-retroviral Therapy (HAART) in clinical practice has improved the survival of people living with HIV (PLHIV) and substantially decreased morbidity and mortality [1]. Life expectancy for PLHIV treated with ART has increased, transforming HIV infection into a chronic condition [2]. Consequently, healthcare expenditure (i.e. drugs, diagnostic exams, specialist visits, hospitalisations) directly related to the management of PLHIV increased [3], both at individual and at population level. This led the payers to design solutions to reduce public healthcare expenditure, in order to maintain the sustainability for the Healthcare Services, at the same time preserving the highest medical care standards.

Recent publications reported that Healthcare Services worldwide called for Clinical Governance (CG) tools to ensure quality and sustainability of HIV treatment and care [4]. In particular, the most relevant strategies were: (i) the rational use of diagnostic testing [5]; (ii) the implementation of health economics approach, in particular the definition of cost-effectiveness criteria [6], to compare the performance of different therapeutic alternatives, in addition to an economic forecast concerning the overall budget devoted to PLHIV treatment and care [7,8]; (iii) the use of protease inhibitor (PI) mono and dual therapies, the administration of Non-Nucleoside Reverse-Transcriptase Inhibitors (NNRTI)-based, instead of PI-based regimens, and the introduction of generic anti-retroviral drugs [5,6,7]. All these strategies could impact on the performances of the Hospitals Units and clinicians, taking in charge PLHIV and dealing with the problem of HIV, modifying the approach to the patients.

In Italy, the access to HIV treatment, related diagnostic exams, specialist visits and hospitalisations, is completely free for the patients and delivered mainly by hospitals, through Infectious Diseases Units. Each Regional Health Service (RHS)–at the decentralised level—is responsible for delivering services for prevention, hospital care and community care, and covers pharmaceutical expenditure for essential categories [9].

Within an Italian local setting, Lombardy Region (the Italian Region with the highest prevalence for HIV infection and a significant HIV incidence rate [10,11]) proposed some CG tools for monitoring and controlling HIV related expenditure. Two main activities were implemented in Lombardy Region: i) direct delivery of drugs by hospitals, providing HIV+ patients with ART, covering a time horizon of no more than 60 days, and ii) introduction of a Clinical Pathway (CP), in August 2011.

Even if the literature evidence debates on the correct definition of a CP, five criteria were generally taken into account for a CP predisposition and implementation: 1) the CP strictly implies a multidisciplinary approach; 2) the CP implementation aims at standardising phases, procedures or episodes of healthcare delivery, within a specific target population [12,13,14]; 3) the pathway translates evidence and guidelines in the different local settings [15]; 4) the CP could be synthesise in a “inventory of actions” [16], to be implemented and measured to monitor the clinicians behaviours; 5) the CP presents timeframes, goals and outcomes.

Thus, the studied HIV CP was defined in accordance with the above mentioned five criteria, detailed in the followings:

1. the multi-disciplinary team involved in the CP predisposition was composed of a RHS delegate, a health economics expert, a hospital pharmacist, two Infectious Disease specialists, one patients’ association delegate. All the representatives were directly selected by the Head of Lombardy Region Pharmaceutical Department, and consensus concerning every decision was achieved with the majority of votes;
2. the CP was addressed to the population with HIV diagnosis. In particular: *i)* first line patients, *ii)* patients needing to switch ART regimen for any clinical reason and for economic factors, and *iii)* subjects experiencing a treatment switch due to the failure of the ongoing therapeutic regimen;
3. the most recent National and International HIV guidelines were taken into consideration, in order to write and update the CP, every 12 months;
4. the CP established the diagnostic procedures (in particular when to provide the HIV Genotypic Resistance test, or CD4+ count), the criteria to start the treatment, and how to select it, for the target population. Furthermore, the HIV CP clearly stated that clinicians should select the less expensive pharmacological treatments, that ensure the same clinical condition, in order to facilitate the correct use of available resources, giving the additional possibility to switch treatment for economic reasons and not only for clinical factors. All these recommendations were consistent with HIV evidence and guidelines;
5. in order to verify the CP adequacy, Lombardy Region should i) plan internal auditing activities in each hospital, in order to verify the prescriptive appropriateness, ii) collect once a year, as regards the previous 12 months, the number of: new patients starting the treatment, patients switching treatment for any reason, subjects experiencing failure, patients treated with high-cost drugs, average monthly and annual cost per Infectious Disease Department, comparing and sharing this information with all Regional Hospitals, using a benchmarking approach; iii) verify every year, with specific performance indicators, the reduction in resources consumption and the effectiveness of the HIV CP implementation.

The introduction of this CP (mandatory since 2011 for all the Lombardy Region Hospitals) was also encouraged as a strategic objective to be achieved by the Hospital General Managers and consequently by the Head of Infectious Diseases Units.

Moving on from these premises, the primary objective of this study was to evaluate the impact of the introduction of Lombardy Region HIV CP in the target population, in terms of patients’ virological and immunological conditions. In particular, the proportions of patients achieving undetectable viral load (VL <50 copies/mL) and CD4+ ≥ 500 was investigated as effectiveness criteria. The secondary objective of the study was to evaluate the overall HIV-related management costs, before and after the CP implementation, considering the budget holder (RHS) perspective, consistently with the criteria previously described, and verifying if the strategic goals defined by the HIV CP were achieved by the hospitals.

## Materials and Methods

### Study design

A multi-centre observational cohort study was designed, composed of two different phases: *i)* the first one was related to the situation before the implementation of the CP (before 2011, called Pre-CP), retrospectively, and *ii)* the second phase concerning the period after the application of the CP (after 2011, called Post-CP), prospectively. The randomisation of the centres was not possible, for ethical and legislative reasons (the implementation of the HIV CP became mandatory in all the regional hospitals in 2011). The only possible study design was to compare the Infectious Disease Departments performances considering the same organisations and clinicians, before and after the CP implementation.

Data from 6 Lombardy Region Hospitals were collected. These Hospitals provided care to approximately 42% of HIV+ patients in the Lombardy Region (Sacco Hospital, Milan; San Raffaele Scientific Institute, Milan; San Paolo Hospital, Milan; Alessandro Manzoni Hospital, Lecco; Ospedale di Circolo Hospital, Busto Arsizio, and Ospedale Civile Hospital, Legnano).

Inclusion criteria were consistent with the target population of HIV CP: all HIV infected adults, who started the first ART (first-line), who switched treatment (for any clinical reasons) or who changed the therapeutic regimen for failure, were enrolled in the study cohort, between November 2009 and November 2010 (Pre-CP phase) and between September 2011 and September 2012 (Post-CP phase).

Data were collected at baseline and after 12-month follow up, in both phases. HIV+ patients were stratified into the three categories defined by the Lombardy Region CP [15], and considered as independent samples of reference for the analysis: (i) “First-line” patients, who started the first ART; (ii) “Switch for toxicity/other” patients, who switched treatment for toxicity, comorbidity, potential drug interaction, simplification (in this category the reduction in the number of pills or reduction in the treatment burden were considered), and also for economic reason, as specifically requested by the HIV CP; (iii) “Switch for failure” patients, who changed treatment because of viro-immunological and/or clinical failure.

The main outcome measures were viro-immunological status and management costs, compared by period (pre- and post-CP) and stratified by patients’ category (First line, Switch for toxicity/other, Switch for failure).

The study was approved by the Hospitals Ethics Committees: the study have been approved by the Ethic Committee of “A.O. Ospedale Lecco”(Coordinating Center) with the following approval number: “Protocollo n. 0016492/12 U 01.02.11 18/04/2012” and informed consent forms were required for all the patients enrolled. The patients provided their written informed consent to participate in this study and the Ethics Committees approved this consent procedure.

### Data collection

The Case Report Forms used for data collection included patients’ demographic information, immunological and virological status, and reasons for treatment modification.

Data related to the economic consumption of resources for the treatment and care of HIV disease such as drugs, hospital admissions and laboratory tests were also collected, using an in-depth analysis of administrative data (i.e. clinical or outpatient records of the Hospital). With regard to economic data, reimbursement tariffs and prices were reported in “euros”, considering the 2014 inflation-rate and values, using the Consumer Price Index for healthcare expenditure where necessary, and making economic measures directly comparable, even considering two different historical periods. The clinical and economic data were recorded at baseline, and then for the following 6 months, both retrospectively in Pre-CP and prospectively in Post-CP phase, for each patient.

### Statistical methods

Categorical variables were described by frequency (%) and compared using the heterogeneity Chi-Square test or the Mantel-Haenszel Chi-Square test, as appropriate; the Cochrane-Mantel-Hanszel test was used to control for potential confounders. Normally distributed continuous variables were described by means (±standard error, SE) and compared using the analysis of variance; a general linear model was applied when to adjust for potential confounding variables. As they were right-skewed, costs were described both by medians (interquartile range, IQR) and means (± SE), and compared using the Wilcoxon-Mann-Whitney test and the analysis of variance. If results of these analyses were inconsistent, the non-parametric test, more conservative, prevailed.

Effectiveness was evaluated at 12-month visit (snapshot analysis) according to intention to treat: patients were considered as first line, switch for toxicity/other, switch for failure, even if during the study period they changed one or more treatments.

All statistical analyses were performed using the statistical software SAS/STAT 9.2 (SAS Institute Inc., Cary, NC, USA).

## Results

### Study sample

The total numbers of patients on ART, taken in charge by the 6 participating hospitals, were 10,079 in Pre-CP phase and 11,143 in Post-CP phase [11].

Patients meeting the inclusion criteria, and stratified for the three categories of interest, were 1,284 in Pre-CP phase and 1,135 in the Post-CP phase.

Patients’ characteristics are reported in Table 1. Most patients switched ART for toxicity or other reasons: the reasons for treatment switch are detailed in Table 2 (and in S1–S4 Tables). Main reasons were toxicity (49.1% *vs* 49.8%), reduction of number of pills (26.3% *vs* 23.6%) and adherence (7.4% *vs* 7.2%). The category of switch called “CP indication” was an economic reason for treatment change, required for chose the less costly drugs regimen, to be considered only in the Post-CP Scenario, because not previously present: it represented only the 3.8% of the switches total amount.

**Table 1:** Patients’ baseline characteristics, Lombardy Region, Italy, comparing Pre-CP (2009–2010) and Post-CP (2011–2012).

	Pre-CP (n = 1,284)		Post-CP (n = 1,135)		
	N	%	N	%	*p-value*
***CP Category***					
*First Line*	324	25.23	268	23.61	**0.002**
*Switch for toxicity/other*	733	57.09	717	63.17	
*Switch for failure*	227	17.68	150	13.22	
***Gender (overall population)***					
*M*	965	75.16	844	74.36	**0.65**
*F*	319	24.84	291	25.64	
***Age (overall population)***					
*<30*	42	3.3	59	5.2	**0.68**
*30–39*	261	20.3	205	18.1	
*40–49*	570	44.4	476	41.9	
*50–59*	293	22.8	286	25.2	
*> = 60*	118	9.2	109	9.6	
***Risk factor for HIV acquisition (overall population)***					
*Drugs Abuse*	277	21.6	238	21.0	**<0.0001**
*Heterosexual*	398	31.0	356	31.4	
*Homosexual*	351	27.3	311	27.4	
*Other*	94	7.3	223	19.6	
*Unknown*	164	12.8	7	0.6	
***ART initiation (overall population)***					
*until 1995*	171	13.3	145	12.8	**0.39**
*1996 to 2005*	543	42.3	448	39.5	
*2006-study start*	567	44.2	526	46.3	
*Unknown*	3	0.2	16	1.4	
***HBV and/or HCV co-infection (overall population)***					
*Yes*	306	23.8	290	25.6	**0.33**
***Most frequent ART therapies (overall population)***					
*Abacavir*	160	12.5	223	19.6	**<0.0001**
*Lamivudine*	283	22.0	316	27.8	**0.001**
*Zidovudine*	63	4.9	40	3.5	**0.09**
*Tenofovir*	810	63.1	639	56.3	**0.0007**
*Emtricitabine*	794	61.8	627	55.2	**0.001**
*Raltegravir*	182	14.2	121	10.7	**0.009**
*Maraviroc*	50	3.9	30	2.6	**0.09**
*Lopinavir*	126	9.8	92	8.1	**0.14**
*Darunavir*	244	19.0	293	25.8	**<0.0001**
*Atazanavir*	422	32.9	332	29.2	**0.055**
*Nevirapine*	94	7.3	136	12.0	**<0.0001**
*Efavirenz*	270	21.0	182	16.0	**0.002**
**First line**					
*Abacavir*	20	6.2	25	9.3	**0.15**
*Lamivudine*	36	11.1	41	15.3	**0.13**
*Zidovudine*	16	4.9	14	5.2	**0.87**
*Tenofovir*	280	86.4	221	82.5	**0.18**
*Emtricitabine*	279	86.1	220	82.1	**0.18**
*Raltegravir*	17	5.3	8	3.0	**0.17**
*Maraviroc*	9	2.8	2	0.8	**0.07**
*Lopinavir*	31	9.6	21	7.8	**0.46**
*Darunavir*	88	27.2	73	27.2	**0.98**
*Atazanavir*	76	23.5	72	26.9	**0.34**
*Nevirapine*	14	4.3	13	4.8	**0.76**
*Efavirenz*	103	31.8	85	31.7	**0.98**
**Switch for toxicity**					
*Abacavir*	117	16.0	184	25.7	**<0.0001**
*Lamivudine*	197	26.9	244	34.0	**0.003**
*Zidovudine*	36	4.9	19	2.6	**0.02**
*Tenofovir*	411	56.1	336	46.9	**0.0005**
*Emtricitabine*	405	55.2	334	46.6	**0.001**
*Raltegravir*	114	15.6	79	11.0	**0.01**
*Maraviroc*	13	1.8	14	1.9	**0.80**
*Lopinavir*	69	9.4	56	7.8	**0.28**
*Darunavir*	85	11.6	152	21.2	**<0.0001**
*Atazanavir*	273	37.2	220	30.7	**0.008**
*Nevirapine*	75	10.2	117	16.3	**0.0006**
*Efavirenz*	144	19.6	93	13.0	**0.0006**
**Switch for failure**					
*Abacavir*	23	10.1	14	9.3	**0.80**
*Lamivudine*	50	22.0	31	20.7	**0.75**
*Zidovudine*	11	4.8	7	4.7	**0.94**
*Tenofovir*	119	52.4	82	54.7	**0.67**
*Emtricitabine*	110	48.5	73	48.7	**0.97**
*Raltegravir*	51	22.5	34	22.7	**0.96**
*Maraviroc*	28	12.3	14	9.3	**0.36**
*Lopinavir*	26	11.4	15	10.0	**0.66**
*Darunavir*	71	31.3	68	45.3	**0.006**
*Atazanavir*	73	32.2	40	26.7	**0.25**
*Nevirapine*	5	2.2	6	4.0	**0.31**
*Efavirenz*	23	10.1	4	2.7	**0.007**

**Table 2:** Reasons for therapy switch at baseline in patients who switched for Toxicity/other, Lombardy Region, Italy, comparing Pre-CP (2009–2010) and Post-CP (2011–2012).

Reason for therapy switch	Pre-CP (N = 733)		Post-CP (N = 717)		*p-value*
	N	%	N	%	
*Toxicitiy*	360	49.1	357	49.8	**<0.0001**
*Simplification*	193	26.3	169	23.6	
*Adherence*	54	7.4	52	7.2	
*Comorbidities*	36	4.9	16	2.2	
*Patient's choice*	33	4.5	24	3.4	
*Drug-drug interaction*	11	1.5	14	2.0	
*CP indication*	0	0.00	27	3.8	
*Other*	46	6.3	58	8.1	

In both samples, patients were predominantly males. Study population was 46.4 ±0.2 years old, the mean age was 46.3 ±0.3 years old in Pre-CP Phase and 46.5 ±0.3 years old in the Post-CP Phase; these data were consistent with the general HIV+ population taken in charge by the Italian National Healthcare Service [17].

The main risk factors for HIV acquisition were heterosexual intercourse for women and homosexual intercourse for men.

On average, at the baseline, the study population have been receiving anti-retroviral therapy for approximately 8.2 ±0.1 years (8.1 ±0.2 years in Pre-CP Phase *vs* 8.2 ±0.2 years in Post-CP Phase).

With regard to ART, proportions of PI-based and NNRTI-based therapies were similar in Pre-CP and Post CP phases (PI: 64.3% vs 64.9%; NNRTI: 30.6% vs 32.0% respectively; p>0.05). On the contrary, Raltegravir was prescribed as a part of the ART regimen more frequently in the Pre-CP (14.3%) than in Post-CP (10.8%) phase (p = 0.008), as well as Maraviroc (3.9% vs 2.7%, p = 0.11). These results are consistent with the treatment indications of HIV CP and the overall achievement of economic objectives of cost monitoring: HIV CP discouraged the Raltegravir and Maraviroc prescription, only for expensiveness reasons and only if an alternative therapeutic regimen could guarantee to the patients the same level of effectiveness, without modifying the expected clinical outcome, paying attention to the presence of eventual resistances.

### Effectiveness results

Undetectable HIV-RNA level at 12-month follow up was achieved by 81.2% of patients in Pre-CP phase, and 83.2% in Post-CP (Table 3). No statistically significant differences emerged, comparing Pre-CP and Post-CP (p = 0.12). However, comparing groups by category, 86.9% of Post-CP “First Line” population achieved undetectable HIV-RNA at 12 months, *versus* 79.4% in Pre-CP phase (p = 0.002).

**Table 3:** Patients with undetectable HIV-RNA at 12-month follow up, Lombardy Region, Italy, comparing Pre-CP (2009–2010) and Post-CP (2011–2012).

Undetectable HIV RNA	Pre-CP (n = 1,284)		Post-CP (n = 1,135)		*p-value*
	N	% (95% CI)	N	% (95% CI)	
*Study population*	1,043	81.2 (79.0–83.3)	944	83.2 (80.9–85.2)	**0.12**
*First Line*	257	79.3 (74.6–83.4)	233	86.9 (82.4–90.5)	**0.002**
*Switch for toxicity/other*	636	86.8 (84.1–89.0)	611	85.2 (88.4–87.6)	**0.64**
*Switch for failure*	150	66.2 (59.7–71.9)	100	66.7 (58.8–73.7)	**0.91**

Mean Nadir CD4^+^ cell counts were 221 ±5 cells/mm^3^ in Pre-CP and 233 ±5 cells/mm^3^ in Post-CP Phase, respectively. In the overall population, Nadir CD4^+^ cell counts were 227 ± 3 cells/mm^3^.

At baseline, CD4+ cell counts were slightly lower in Pre-CP than in Post-CP population (510 ± 9.5 vs 537 ± 8 respectively, p = 0.02). Mean change from baseline was +28 ± 4 in Pre-CP and +39 ± 5 in Post-CP (p = 0.11). As a result, at follow up CD4+ cell counts were still lower in Pre-CP phase (539 ± 8 vs 581 ± 9 in Post-CP phase, p = 0.0004). Results by CD4+ class are given in Table 4. Performing the analyses by category, a significantly higher recovery was found in the “First line” group (Table 5), whereas no difference emerged in other groups and in the whole sample, even when including the baseline CD4+ value in the analysis equation.

**Table 4:** CD4+ distribution in the study population, at baseline and 12-month follow up, Lombardy Region, Italy, comparing Pre-CP (2009–2010) and Post-CP (2011–2012).

CD4+ cells/μL	Pre-CP				Post-CP			
	Baseline (N = 1253)		Follow-up (N = 1227)		Baseline (N = 1079)		Follow-up (N = 1026)	
	N	% (95% CI)	N	% (95% CI)	N	% (95% CI)	N	% (95% CI)
≤ 200	137	10.9 (9.3–12.8)	107	8.7 (7.3–10.4)	104	9.6 (8.0–11.6)	72	7.0 (5.6–8.7)
201–350	214	17.1 (15.1–19.3)	201	16.4 (14.4–18.6)	178	16.5 (14.4–18.8)	143	13.9 (12.0–16.2)
351–499	318	25.4 (23.-27.9)	284	23.2 (20.9–25.6)	230	21.3 (19.0–23.9)	213	20.8 (18.4–23.4)
≥ 501	584	46.6 (43.8–49.2)	635	51.8 (49.0–54.5)	567	52.6 (49.6–55.5)	598	58.3 (55.2–61.3)

Baseline comparison (entire cohorts, considered as the sum of the three HIV CP categories) Pre-CP vs Post-CP p-value = 0.04.

Follow-up comparison (entire cohorts, considered as the sum of the three HIV CP categories): Pre-CP vs Post-CP p-value = 0.003; controlled for category: p-value = 0.02; controlled for initial CD4+ level: p-value = 0.05; controlled for category and initial CD4+ level: p-value = 0.066.

**Table 5:** Mean CD4+ change from baseline (12-month follow-up—baseline), Lombardy Region, Italy, comparing Pre-CP (2009–2010) and Post-CP (2011–2012).

CD4+ count [cells/μL]	Pre-CP (n = 1,207)	Post-CP (n = 1,008)	*p-value*
*Study population*	28 ± 4	39 ± 5	0.11
*First Line*	36 ± 9	70 ± 11	0.02
*Switch for toxicity/other*	22 ± 6	26 ± 6	0.62
*Switch for failure*	38 ± 10	45 ± 12	0.67

In conclusion, effectiveness results (Tables 3, 4 and 5) showed that the same level of virological and immunological effectiveness was equally guaranteed to the HIV+ patients in the two compared periods, thus being consistent with the indication of HIV CP (i.e. the selection of the less expensive pharmacological treatments, ensuring the same clinical condition).

### Costs results

All the economic and costs analysis results are presented as “per-patient” observation.

As showed in Table 6, both mean and median costs were lower in the Post-CP than in Pre-CP phase. After CP implementation, ART expenditure decreased by 8.7% as compared to Pre-CP. The greatest reduction was in the “Switch for toxicity/others” group (-9.9%), followed by the “Switch for failure” group (-6.1%) and by the “First Line” group (-4.0%).

**Table 6:** Mean yearly per-patient ART costs, Lombardy Region, Italy, comparing Pre-CP (2009–2010) and Post-CP (2011–2012), related to 2014 price level.

ART cost	Pre-CP (€) Mean±SE Median (IQR)	Post-CP (€) Mean±SE Median (IQR)	*p-value*	Absolute Value (€) Pre-CP vs Post-CP scenario	% Pre-CP vs Post-CP scenario
*Study Population*	9,540.24 ± 96.20 9,601.28 (7,948.76–10,039.70)	8,708.28 ± 91.58 9,199.56 (7,629.39–9,877.83)	**<0.0001**	-831.96	-8.72%
*First Line*	9,230.49 ± 148.50 9,485.96 (7,948.76–9,885.69)	8,863.48 ± 97.76 9,275.84 (7,948.76–9,694.44)	**0.26**	-367.01	-3.98%
*Switch for toxicity/other*	9,223.73 ± 118.77 9,199.56 (7,799.31–10,656.67)	8,309.78 ± 114.08 8,180.69 (6,510.91–9,694.44)	**<0.0001**	-913.95	-9.91%
*Switch for failure*	11,004.38 ± 305.14 9,694.44 (8,099.74–12,465.65)	10,335.83 ± 360.93 9,694.44 (8,050.25–12,371.11)	**0.36**	-668.55	-6.08%

As showed in Table 7, considering total costs of HIV disease management (ART, hospital admissions and laboratory tests), in Post-CP Phase the mean total costs decreased by -8.6%. As for ART costs, the greatest reduction was seen in the “Switch for toxicity/other” group. This economic result is more consistent considering that Pre and Post CP analysis were performed with the same pricing and reimbursement level, related to the year 2014.

**Table 7:** Mean yearly per-patient total costs, Lombardy Region, Italy, comparing Pre-CP (2009–2010) and Post-CP (2011–2012), evaluated with 2014 price and reimbursement tariffs.

Total costs*	Pre-CP (€) Mean±SE Median (IQR)	Post-CP (€) Mean±SE Median (IQR)	*p-value*	Absolute Value (€) Pre-CP vs Post-CP scenario	% Pre-CP vs Post-CP scenario
*Study Population*	10,130.85 ± 111.95 9,800.03 (8,089.89–11,080.00)	9,259.74 ± 107.31 9,424.63 (7,778.45–10,126.54)	**<0.0001**	-871.11	-8.60%
*First Line*	9,955.81 ± 185.49 9,773.31 (8,190.68–10,129.34)	9,505.09 ± 157.03 9,589.69 (8,168.09–10,023.04)	**0.22**	-450.72	-4.53%
*Switch for toxicity/other*	9,783.16 ± 144.31 9,606.69 (8,003.31–11,109.30)	8,827.97 ± 135.34 9,135.43 (6,901.26–10,040.77)	**<0.0001**	-955.19	-9.76%
*Switch for failure*	11,503.41 ± 322.81 9,981.68 (8,516.96–13,461.48)	10,885.20 ± 372.95 9,885.69 (8,479.71–13,094.28)	**0.39**	-618.21	-5.37%

* ART + hospital admission + laboratory tests.

## Discussion

In this study the main finding was that the Lombardy Region HIV CP led to a cost reduction in a 12-month perspective. Consistently with the literature [5–7], in this study we found that not only ART costs, but also hospital admission and laboratory tests costs diminished (-8.6%). This decrease was mainly due to two principal factors: *i)* higher appropriateness in the ART strategies selection and prescription, in accordance with the categories defined by the HIV CP (ART cost savings impacted from the 90 to 95% of the overall cost reduction); *ii)* higher attention in the request of hospitalisations and exam prescription (in particular hospitalisations impacted on the decrease of costs for a percentage from 3 to 6%). This decrease could be interpreted as the result of the internal audit and monitoring system requested by the HIV CP implementation. Infectious Disease Department Directors’ and clinicians’ behaviours changed after the HIV CP implementation, but not in clinical and effectiveness terms. They kept on prescribing and taking decisions according to recommendations and the guidelines indications, but with a greater focus on resources consumption and costs containment factors. The costs reduction in the study population was not dependent on the discount applied in the drugs prices, but on a more homogeneous prescribing behaviour. The statistically significant reduction in the “Switch for toxicity/other” population could be partially due to the impact of CP switches, induced by economic reasons.

The CP was developed by the clinicians of reference at regional level, updated and verified every 12 months, following National and International guidelines concerning this topic, thus establishing an evidence-based methodology. This kind of collaborative approach, among clinicians, pharmacists and decision makers, was able to create a profound commitment and common language, useful also for a rationalisation of resources in HIV disease management.

Disinvestment and costs reduction are always priorities for the policy and decision makers agendas, but in particular in the current era of economic recession. Strategies are needed to reduce costs, preserving effectiveness and appropriateness, and guaranteeing universality and equity of care in the National Healthcare Services. An appropriate approach, in ART prescription, may reduce the healthcare expenditure and the related budget. However, a more attentive implementation and application of the International and National guidelines’ requirements is needed to achieve appropriateness criteria at local level. Only this “real” world and local setting become the more suitable place for the evaluation of the decision making process, in terms of efficiency and effectiveness of the HIV management.

Considering the HIV CP impact in terms of efficiency, despite the constant increase in the number of patients, findings suggest that CP increased the efficiency of resources allocation, guaranteeing the same immunological and virological outcomes.

Other strategies reported in literature [5, 7, 8] useful to ensure economic sustainability of HIV treatment and care, did not assess specific efficacy measures. This could be considered the significant contribution of this study: for the implementation of a proper decision making process, a multi-dimensional approach based on both outputs produced and outcomes guaranteed to patients is required.

Potential limitations of the study should be mentioned. The generalisation of results and conclusions from this study to other local settings should be conducted with caution because the used cohort data may reflect the specificity of a particular setting of care. Moreover, the study did not take into consideration the costs related to the drugs administered to patients for the treatment of HIV adverse events or other co-morbidities that were prescribed by General Practitioners, outside the hospital setting. Lastly, we just considered a 12-month time span: long-term consequences of these new indications remain to be assessed.

Despite these limitations, the results show the presence of a good “real” world practice in Italy. The Lombardy Region HIV CP in the study period preserved the immunological, virological and clinical status of patients, with a positive economic impact in terms of costs saving for treatments, hospital admissions and laboratory tests.

In conclusion, the findings of the study may have important implications for decision makers, not only in Italy, but also in other countries. The implementation of a CP, that incorporated appropriateness and economic criteria on the basis of National and International HIV guidelines, could be considered an effective Clinical Governance tool. Of course, local peculiarities (in terms of organisational settings and financial capacity) need to be taken into account.

Given the growing literature on the impact of Clinical Governance tools, the present study contributes to provide reliable data, on the cost reduction of HIV disease treatment and care, with no loss of opportunity for HIV+ patients in terms of efficacy and quality of care.

## Supporting Information

S1 Table HAART regimen—Overall. (DOCX)

S2 Table HAART regimen by CP category: *category = 1*. first line therapy.
(DOCX)

S3 Table HAART regimen by CP category: category = 2. Toxicity/other.
(DOCX)

S4 Table HAART regimen by CP category: category = 3. Failure.
(DOCX)
