# Supplementary material for: HIV Clinical Pathway: A New Approach to Combine Guidelines and Sustainability of Anti-Retroviral Treatment in Italy
Source: PLoS One. 2016 Dec 28;11(12):e0168399. doi: 10.1371/journal.pone.0168399 (PMC5193418; doi:10.1371/journal.pone.0168399)
Supplement: S1 Table — (DOCX) [file pone.0168399.s001.docx]

**S1 Table. HAART regimen - Overall**

| *PERIOD* | *HAART* | *Frequency* | *Percent* | *Cumulative Frequency* | *Cumulative Percent* |
| --- | --- | --- | --- | --- | --- |
| *1. PRE-CP* | *3TC* | 4 | 0.17 | 4 | 0.17 |
| *1. PRE-CP* | *3TC + ABC + ATV + RTV* | 4 | 0.17 | 8 | 0.33 |
| *1. PRE-CP* | *3TC + ATV* | 4 | 0.17 | 12 | 0.50 |
| *1. PRE-CP* | *3TC + ATV + MVC* | 1 | 0.04 | 13 | 0.54 |
| *1. PRE-CP* | *3TC + ATV + RAL* | 4 | 0.17 | 17 | 0.70 |
| *1. PRE-CP* | *3TC + ATV + RAL + RTV* | 3 | 0.12 | 20 | 0.83 |
| *1. PRE-CP* | *3TC + ATV + RTV* | 5 | 0.21 | 25 | 1.03 |
| *1. PRE-CP* | *3TC + ATV + RTV + TDF* | 4 | 0.17 | 29 | 1.20 |
| *1. PRE-CP* | *3TC + ATV + TDF* | 1 | 0.04 | 30 | 1.24 |
| *1. PRE-CP* | *3TC + ATV + ddl* | 3 | 0.12 | 33 | 1.36 |
| *1. PRE-CP* | *3TC + AZT + DRV + RTV* | 1 | 0.04 | 34 | 1.41 |
| *1. PRE-CP* | *3TC + AZT + RAL* | 1 | 0.04 | 35 | 1.45 |
| *1. PRE-CP* | *3TC + DRV + ATV + RTV* | 2 | 0.08 | 37 | 1.53 |
| *1. PRE-CP* | *3TC + DRV + EFV + RAL + RTV* | 1 | 0.04 | 38 | 1.57 |
| *1. PRE-CP* | *3TC + DRV + EFV + RTV* | 1 | 0.04 | 39 | 1.61 |
| *1. PRE-CP* | *3TC + DRV + ETV + RTV* | 3 | 0.12 | 42 | 1.74 |
| *1. PRE-CP* | *3TC + DRV + RAL + RTV* | 3 | 0.12 | 45 | 1.86 |
| *1. PRE-CP* | *3TC + DRV + RTV* | 3 | 0.12 | 48 | 1.98 |
| *1. PRE-CP* | *3TC + DRV + RTV + TDF* | 1 | 0.04 | 49 | 2.03 |
| *1. PRE-CP* | *3TC + EFV+ RAL* | 1 | 0.04 | 50 | 2.07 |
| *1. PRE-CP* | *3TC + FPV* | 1 | 0.04 | 51 | 2.11 |
| *1. PRE-CP* | *3TC + FPV + RTV + TDF* | 1 | 0.04 | 52 | 2.15 |
| *1. PRE-CP* | *3TC + LPV/r* | 3 | 0.12 | 55 | 2.27 |
| *1. PRE-CP* | *3TC + LPV/r + TDF* | 1 | 0.04 | 56 | 2.32 |
| *1. PRE-CP* | *3TC + NVP + RAL* | 2 | 0.08 | 58 | 2.40 |
| *1. PRE-CP* | *3TC + RAL* | 3 | 0.12 | 61 | 2.52 |
| *1. PRE-CP* | *3TC + RAL + NVP + MVC* | 1 | 0.04 | 62 | 2.56 |
| *1. PRE-CP* | *3TC + RAL + TDF* | 1 | 0.04 | 63 | 2.60 |
| *1. PRE-CP* | *3TC + RTV + ATV + RAL* | 1 | 0.04 | 64 | 2.65 |
| *1. PRE-CP* | *3TC + STV + DRV + ETV + MVC + RTV + T20* | 1 | 0.04 | 65 | 2.69 |
| *1. PRE-CP* | *3TC + STV + RAL* | 1 | 0.04 | 66 | 2.73 |
| *1. PRE-CP* | *3TC + TDF* | 1 | 0.04 | 67 | 2.77 |
| *1. PRE-CP* | *3TC + ddl* | 1 | 0.04 | 68 | 2.81 |
| *1. PRE-CP* | *3TC + ddl + DRV + RTV* | 2 | 0.08 | 70 | 2.89 |
| *1. PRE-CP* | *3TC + ddl + MVC* | 1 | 0.04 | 71 | 2.94 |
| *1. PRE-CP* | *3TC + ddl + NVP* | 1 | 0.04 | 72 | 2.98 |
| *1. PRE-CP* | *3TC/ABC* | 2 | 0.08 | 74 | 3.06 |
| *1. PRE-CP* | *3TC/ABC + ATV* | 39 | 1.61 | 113 | 4.67 |
| *1. PRE-CP* | *3TC/ABC + ATV + LPV/r* | 1 | 0.04 | 114 | 4.71 |
| *1. PRE-CP* | *3TC/ABC + ATV + NVP* | 1 | 0.04 | 115 | 4.75 |
| *1. PRE-CP* | *3TC/ABC + ATV + RAL + RTV* | 1 | 0.04 | 116 | 4.80 |
| *1. PRE-CP* | *3TC/ABC + ATV + RTV* | 35 | 1.45 | 151 | 6.24 |
| *1. PRE-CP* | *3TC/ABC + DRV + RAL + RTV* | 1 | 0.04 | 152 | 6.28 |
| *1. PRE-CP* | *3TC/ABC + DRV + RTV* | 13 | 0.54 | 165 | 6.82 |
| *1. PRE-CP* | *3TC/ABC + EFV* | 15 | 0.62 | 180 | 7.44 |
| *1. PRE-CP* | *3TC/ABC + ETV* | 1 | 0.04 | 181 | 7.48 |
| *1. PRE-CP* | *3TC/ABC + ETV + RAL* | 1 | 0.04 | 182 | 7.52 |
| *1. PRE-CP* | *3TC/ABC + FPV + RTV* | 1 | 0.04 | 183 | 7.57 |
| *1. PRE-CP* | *3TC/ABC + LPV/r* | 13 | 0.54 | 196 | 8.10 |
| *1. PRE-CP* | *3TC/ABC + MVC* | 1 | 0.04 | 197 | 8.14 |
| *1. PRE-CP* | *3TC/ABC + NVP* | 19 | 0.79 | 216 | 8.93 |
| *1. PRE-CP* | *3TC/ABC + RAL* | 5 | 0.21 | 221 | 9.14 |
| *1. PRE-CP* | *3TC/ABC + RTV* | 1 | 0.04 | 222 | 9.18 |
| *1. PRE-CP* | *3TC/ABC + TDF + ATV* | 1 | 0.04 | 223 | 9.22 |
| *1. PRE-CP* | *3TC/ABC/AZT* | 4 | 0.17 | 227 | 9.38 |
| *1. PRE-CP* | *3TC/ABC/AZT + FPV + RTV* | 1 | 0.04 | 228 | 9.43 |
| *1. PRE-CP* | *3TC/AZT* | 3 | 0.12 | 231 | 9.55 |
| *1. PRE-CP* | *3TC/AZT + ATV* | 5 | 0.21 | 236 | 9.76 |
| *1. PRE-CP* | *3TC/AZT + ATV + RTV* | 12 | 0.50 | 248 | 10.25 |
| *1. PRE-CP* | *3TC/AZT + DRV + RAL + RTV* | 1 | 0.04 | 249 | 10.29 |
| *1. PRE-CP* | *3TC/AZT + DRV + RTV* | 5 | 0.21 | 254 | 10.50 |
| *1. PRE-CP* | *3TC/AZT + EFV* | 4 | 0.17 | 258 | 10.67 |
| *1. PRE-CP* | *3TC/AZT + FPV + RTV* | 1 | 0.04 | 259 | 10.71 |
| *1. PRE-CP* | *3TC/AZT + LPV/r* | 11 | 0.45 | 270 | 11.16 |
| *1. PRE-CP* | *3TC/AZT + LPV/r + MVC* | 1 | 0.04 | 271 | 11.20 |
| *1. PRE-CP* | *3TC/AZT + NVP* | 8 | 0.33 | 279 | 11.53 |
| *1. PRE-CP* | *3TC/AZT + RAL* | 3 | 0.12 | 282 | 11.66 |
| *1. PRE-CP* | *3TC/AZT + RTV + SQV* | 1 | 0.04 | 283 | 11.70 |
| *1. PRE-CP* | *ATV* | 8 | 0.33 | 291 | 12.03 |
| *1. PRE-CP* | *ATV + FTC + RAL* | 2 | 0.08 | 293 | 12.11 |
| *1. PRE-CP* | *ATV + FTC + RAL + RTV* | 1 | 0.04 | 294 | 12.15 |
| *1. PRE-CP* | *ATV + MVC + RAL* | 2 | 0.08 | 296 | 12.24 |
| *1. PRE-CP* | *ATV + MVC + RAL + RTV* | 2 | 0.08 | 298 | 12.32 |
| *1. PRE-CP* | *ATV + MVC + RTV* | 1 | 0.04 | 299 | 12.36 |
| *1. PRE-CP* | *ATV + MVC + RTV + TDF* | 2 | 0.08 | 301 | 12.44 |
| *1. PRE-CP* | *ATV + MVC + RTV + TDF/FTC* | 3 | 0.12 | 304 | 12.57 |
| *1. PRE-CP* | *ATV + RAL* | 26 | 1.07 | 330 | 13.64 |
| *1. PRE-CP* | *ATV + RAL + RTV* | 1 | 0.04 | 331 | 13.68 |
| *1. PRE-CP* | *ATV + RAL + RTV + TDF* | 1 | 0.04 | 332 | 13.72 |
| *1. PRE-CP* | *ATV + RAL + TDF/FTC* | 1 | 0.04 | 333 | 13.77 |
| *1. PRE-CP* | *ATV + RTV* | 27 | 1.12 | 360 | 14.88 |
| *1. PRE-CP* | *ATV + RTV + ETV* | 1 | 0.04 | 361 | 14.92 |
| *1. PRE-CP* | *ATV + RTV + FVP* | 1 | 0.04 | 362 | 14.96 |
| *1. PRE-CP* | *ATV + RTV + TDF/FTC* | 180 | 7.44 | 542 | 22.41 |
| *1. PRE-CP* | *ATV + TDF/FTC* | 30 | 1.24 | 572 | 23.65 |
| *1. PRE-CP* | *ATV + ddl* | 1 | 0.04 | 573 | 23.69 |
| *1. PRE-CP* | *ATV + ddl + NVP + RTV* | 1 | 0.04 | 574 | 23.73 |
| *1. PRE-CP* | *ATV + ddl + RTV + TDF* | 1 | 0.04 | 575 | 23.77 |
| *1. PRE-CP* | *ATV+SQV* | 1 | 0.04 | 576 | 23.81 |
| *1. PRE-CP* | *DRV + EFV + FTC + RTV* | 1 | 0.04 | 577 | 23.85 |
| *1. PRE-CP* | *DRV + EFV + RAL + RTV* | 1 | 0.04 | 578 | 23.89 |
| *1. PRE-CP* | *DRV + ETV + MVC + RAL + RTV* | 1 | 0.04 | 579 | 23.94 |
| *1. PRE-CP* | *DRV + ETV + MVC + RTV* | 1 | 0.04 | 580 | 23.98 |
| *1. PRE-CP* | *DRV + ETV + RAL + RTV* | 3 | 0.12 | 583 | 24.10 |
| *1. PRE-CP* | *DRV + ETV + RAL + RTV + TDF/FTC* | 1 | 0.04 | 584 | 24.14 |
| *1. PRE-CP* | *DRV + ETV + RTV* | 2 | 0.08 | 586 | 24.22 |
| *1. PRE-CP* | *DRV + ETV + RTV + TDF* | 1 | 0.04 | 587 | 24.27 |
| *1. PRE-CP* | *DRV + MVC + RAL + RTV* | 9 | 0.37 | 596 | 24.64 |
| *1. PRE-CP* | *DRV + MVC + RTV* | 5 | 0.21 | 601 | 24.84 |
| *1. PRE-CP* | *DRV + MVC + RTV + TDF/FTC* | 2 | 0.08 | 603 | 24.93 |
| *1. PRE-CP* | *DRV + RAL + RTV* | 20 | 0.83 | 623 | 25.75 |
| *1. PRE-CP* | *DRV + RAL + RTV + TDF* | 3 | 0.12 | 626 | 25.88 |
| *1. PRE-CP* | *DRV + RAL + RTV + TDF/FTC* | 6 | 0.25 | 632 | 26.13 |
| *1. PRE-CP* | *DRV + RTV* | 16 | 0.66 | 648 | 26.79 |
| *1. PRE-CP* | *DRV + RTV + TDF* | 1 | 0.04 | 649 | 26.83 |
| *1. PRE-CP* | *DRV + RTV + TDF + MVC* | 1 | 0.04 | 650 | 26.87 |
| *1. PRE-CP* | *DRV + RTV + TDF/FTC* | 125 | 5.17 | 775 | 32.04 |
| *1. PRE-CP* | *DRV + TDF/FTC* | 3 | 0.12 | 778 | 32.16 |
| *1. PRE-CP* | *EFV* | 1 | 0.04 | 779 | 32.20 |
| *1. PRE-CP* | *EFV + RAL* | 1 | 0.04 | 780 | 32.24 |
| *1. PRE-CP* | *EFV + TDF/FTC* | 44 | 1.82 | 824 | 34.06 |
| *1. PRE-CP* | *ETV + MVC + RAL* | 6 | 0.25 | 830 | 34.31 |
| *1. PRE-CP* | *ETV + MVC + TDF* | 1 | 0.04 | 831 | 34.35 |
| *1. PRE-CP* | *ETV + RAL* | 5 | 0.21 | 836 | 34.56 |
| *1. PRE-CP* | *ETV + RAL + TDF/FTC* | 1 | 0.04 | 837 | 34.60 |
| *1. PRE-CP* | *ETV + TDF/FTC* | 1 | 0.04 | 838 | 34.64 |
| *1. PRE-CP* | *FPV + MVC + RAL + RTV* | 1 | 0.04 | 839 | 34.68 |
| *1. PRE-CP* | *FPV + RAL + TDF/FTC* | 1 | 0.04 | 840 | 34.73 |
| *1. PRE-CP* | *FPV + RTV* | 3 | 0.12 | 843 | 34.85 |
| *1. PRE-CP* | *FPV + RTV + TDF/FTC* | 13 | 0.54 | 856 | 35.39 |
| *1. PRE-CP* | *FPV + TDF/FTC* | 7 | 0.29 | 863 | 35.68 |
| *1. PRE-CP* | *FTC + MVC + RAL* | 1 | 0.04 | 864 | 35.72 |
| *1. PRE-CP* | *LPV/r* | 29 | 1.20 | 893 | 36.92 |
| *1. PRE-CP* | *LPV/r + RAL* | 6 | 0.25 | 899 | 37.16 |
| *1. PRE-CP* | *LPV/r + RAL + TDF/FTC* | 1 | 0.04 | 900 | 37.21 |
| *1. PRE-CP* | *LPV/r + TDF/FTC* | 59 | 2.44 | 959 | 39.64 |
| *1. PRE-CP* | *MVC + ETV* | 1 | 0.04 | 960 | 39.69 |
| *1. PRE-CP* | *MVC + RAL* | 1 | 0.04 | 961 | 39.73 |
| *1. PRE-CP* | *MVC + RAL + T20* | 1 | 0.04 | 962 | 39.77 |
| *1. PRE-CP* | *MVC + TDF/FTC* | 2 | 0.08 | 964 | 39.85 |
| *1. PRE-CP* | *NVP + ATV* | 1 | 0.04 | 965 | 39.89 |
| *1. PRE-CP* | *NVP + DRV + RTV* | 1 | 0.04 | 966 | 39.93 |
| *1. PRE-CP* | *NVP + RAL* | 1 | 0.04 | 967 | 39.98 |
| *1. PRE-CP* | *NVP + TDF/FTC* | 57 | 2.36 | 1024 | 42.33 |
| *1. PRE-CP* | *RAL + T20* | 1 | 0.04 | 1025 | 42.37 |
| *1. PRE-CP* | *RAL + TDF/FTC* | 38 | 1.57 | 1063 | 43.94 |
| *1. PRE-CP* | *T20 + TDF/FTC* | 1 | 0.04 | 1064 | 43.99 |
| *1. PRE-CP* | *TDF + ATV + RTV + AZT* | 1 | 0.04 | 1065 | 44.03 |
| *1. PRE-CP* | *TDF/FTC* | 10 | 0.41 | 1075 | 44.44 |
| *1. PRE-CP* | *TDF/FTC + NVP+ LPV/r* | 1 | 0.04 | 1076 | 44.48 |
| *1. PRE-CP* | *TDF/FTC/EFV* | 200 | 8.27 | 1276 | 52.75 |
| *1. PRE-CP* | *TPV + RTV + MVC + RAL* | 1 | 0.04 | 1277 | 52.79 |
| *1. PRE-CP* | *ddl + DRV + FTC + RTV* | 1 | 0.04 | 1278 | 52.83 |
| *1. PRE-CP* | *ddl + DRV + RAL + RTV* | 2 | 0.08 | 1280 | 52.91 |
| *1. PRE-CP* | *ddl + FTC* | 1 | 0.04 | 1281 | 52.96 |
| *1. PRE-CP* | *sospensione* | 3 | 0.12 | 1284 | 53.08 |
| *2. POST-CP* | *3TC* | 4 | 0.17 | 1288 | 53.25 |
| *2. POST-CP* | *3TC + DRV + RTV* | 1 | 0.04 | 1289 | 53.29 |
| *2. POST-CP* | *3TC + ABC + ATV + RTV* | 1 | 0.04 | 1290 | 53.33 |
| *2. POST-CP* | *3TC + ATV* | 11 | 0.45 | 1301 | 53.78 |
| *2. POST-CP* | *3TC + ATV + RAL* | 2 | 0.08 | 1303 | 53.87 |
| *2. POST-CP* | *3TC + ATV + RTV* | 7 | 0.29 | 1310 | 54.15 |
| *2. POST-CP* | *3TC + ATV + RTV + TDF* | 1 | 0.04 | 1311 | 54.20 |
| *2. POST-CP* | *3TC + ATV + TDF/FTC* | 1 | 0.04 | 1312 | 54.24 |
| *2. POST-CP* | *3TC + ATV+ DRV* | 1 | 0.04 | 1313 | 54.28 |
| *2. POST-CP* | *3TC + AZT + RAL* | 1 | 0.04 | 1314 | 54.32 |
| *2. POST-CP* | *3TC + DRV + NVP + RTV* | 1 | 0.04 | 1315 | 54.36 |
| *2. POST-CP* | *3TC + DRV + RTV* | 8 | 0.33 | 1323 | 54.69 |
| *2. POST-CP* | *3TC + DRV + RTV + TDF* | 3 | 0.12 | 1326 | 54.82 |
| *2. POST-CP* | *3TC + EFV* | 1 | 0.04 | 1327 | 54.86 |
| *2. POST-CP* | *3TC + FPV +RAL* | 1 | 0.04 | 1328 | 54.90 |
| *2. POST-CP* | *3TC + LPV/r* | 5 | 0.21 | 1333 | 55.11 |
| *2. POST-CP* | *3TC + LPV/r + TDF* | 2 | 0.08 | 1335 | 55.19 |
| *2. POST-CP* | *3TC + MVC + LVP/r* | 1 | 0.04 | 1336 | 55.23 |
| *2. POST-CP* | *3TC + MVC + RAL* | 1 | 0.04 | 1337 | 55.27 |
| *2. POST-CP* | *3TC + NVP + RAL* | 2 | 0.08 | 1339 | 55.35 |
| *2. POST-CP* | *3TC + NVP + TDF* | 2 | 0.08 | 1341 | 55.44 |
| *2. POST-CP* | *3TC + ddl + DRV + RTV* | 1 | 0.04 | 1342 | 55.48 |
| *2. POST-CP* | *3TC + ddl + EFV* | 1 | 0.04 | 1343 | 55.52 |
| *2. POST-CP* | *3TC + ddl + LPV/r* | 1 | 0.04 | 1344 | 55.56 |
| *2. POST-CP* | *3TC + ddl + NVP* | 1 | 0.04 | 1345 | 55.60 |
| *2. POST-CP* | *3TC/ABC* | 8 | 0.33 | 1353 | 55.93 |
| *2. POST-CP* | *3TC/ABC + ATV* | 15 | 0.62 | 1368 | 56.55 |
| *2. POST-CP* | *3TC/ABC + ATV + RTV* | 59 | 2.44 | 1427 | 58.99 |
| *2. POST-CP* | *3TC/ABC + AZT* | 1 | 0.04 | 1428 | 59.03 |
| *2. POST-CP* | *3TC/ABC + DRV + RAL + RTV* | 1 | 0.04 | 1429 | 59.07 |
| *2. POST-CP* | *3TC/ABC + DRV + RTV* | 24 | 0.99 | 1453 | 60.07 |
| *2. POST-CP* | *3TC/ABC + DRV + RTV + TDF* | 1 | 0.04 | 1454 | 60.11 |
| *2. POST-CP* | *3TC/ABC + EFV* | 18 | 0.74 | 1472 | 60.85 |
| *2. POST-CP* | *3TC/ABC + ETV* | 8 | 0.33 | 1480 | 61.18 |
| *2. POST-CP* | *3TC/ABC + FPV + RTV* | 3 | 0.12 | 1483 | 61.31 |
| *2. POST-CP* | *3TC/ABC + LPV/r* | 13 | 0.54 | 1496 | 61.84 |
| *2. POST-CP* | *3TC/ABC + NVP* | 53 | 2.19 | 1549 | 64.03 |
| *2. POST-CP* | *3TC/ABC + NVP + RAL* | 2 | 0.08 | 1551 | 64.12 |
| *2. POST-CP* | *3TC/ABC + NVP+MVC* | 1 | 0.04 | 1552 | 64.16 |
| *2. POST-CP* | *3TC/ABC + RAL* | 9 | 0.37 | 1561 | 64.53 |
| *2. POST-CP* | *3TC/ABC + SQV + RTV* | 2 | 0.08 | 1563 | 64.61 |
| *2. POST-CP* | *3TC/ABC/AZT* | 2 | 0.08 | 1565 | 64.70 |
| *2. POST-CP* | *3TC/AZT + ATV + RTV* | 9 | 0.37 | 1574 | 65.07 |
| *2. POST-CP* | *3TC/AZT + DRV + RTV* | 2 | 0.08 | 1576 | 65.15 |
| *2. POST-CP* | *3TC/AZT + EFV* | 4 | 0.17 | 1580 | 65.32 |
| *2. POST-CP* | *3TC/AZT + ETV* | 2 | 0.08 | 1582 | 65.40 |
| *2. POST-CP* | *3TC/AZT + LPV/r* | 13 | 0.54 | 1595 | 65.94 |
| *2. POST-CP* | *3TC/AZT + RAL* | 3 | 0.12 | 1598 | 66.06 |
| *2. POST-CP* | *ABC + TDF+ ATV + RTV* | 1 | 0.04 | 1599 | 66.10 |
| *2. POST-CP* | *ATV* | 6 | 0.25 | 1605 | 66.35 |
| *2. POST-CP* | *ATV + TDF/FTC* | 1 | 0.04 | 1606 | 66.39 |
| *2. POST-CP* | *ATV + DRV + RTV + TDF/FTC* | 1 | 0.04 | 1607 | 66.43 |
| *2. POST-CP* | *ATV + ETV + RAL + RTV* | 1 | 0.04 | 1608 | 66.47 |
| *2. POST-CP* | *ATV + FTC* | 1 | 0.04 | 1609 | 66.52 |
| *2. POST-CP* | *ATV + FTC + RTV* | 1 | 0.04 | 1610 | 66.56 |
| *2. POST-CP* | *ATV + MVC + RTV* | 1 | 0.04 | 1611 | 66.60 |
| *2. POST-CP* | *ATV + RAL + RTV* | 5 | 0.21 | 1616 | 66.80 |
| *2. POST-CP* | *ATV + RAL + RTV + TDF* | 1 | 0.04 | 1617 | 66.85 |
| *2. POST-CP* | *ATV + RTV* | 22 | 0.91 | 1639 | 67.76 |
| *2. POST-CP* | *ATV + RTV + FTC + MVC* | 1 | 0.04 | 1640 | 67.80 |
| *2. POST-CP* | *ATV + RTV + TDF/FTC* | 165 | 6.82 | 1805 | 74.62 |
| *2. POST-CP* | *ATV + TDF/FTC* | 12 | 0.50 | 1817 | 75.11 |
| *2. POST-CP* | *ATV+FTC* | 2 | 0.08 | 1819 | 75.20 |
| *2. POST-CP* | *AZT + ATV + RAL* | 1 | 0.04 | 1820 | 75.24 |
| *2. POST-CP* | *AZT + TDF/FTC* | 1 | 0.04 | 1821 | 75.28 |
| *2. POST-CP* | *DRV + ETV + MVC + RTV* | 1 | 0.04 | 1822 | 75.32 |
| *2. POST-CP* | *DRV + ETV + RAL + RTV* | 5 | 0.21 | 1827 | 75.53 |
| *2. POST-CP* | *DRV + ETV + RAL + RTV + TDF/FTC* | 1 | 0.04 | 1828 | 75.57 |
| *2. POST-CP* | *DRV + ETV + RTV* | 16 | 0.66 | 1844 | 76.23 |
| *2. POST-CP* | *DRV + ETV + RTV + TDF* | 1 | 0.04 | 1845 | 76.27 |
| *2. POST-CP* | *DRV + MVC + RAL + RTV* | 2 | 0.08 | 1847 | 76.35 |
| *2. POST-CP* | *DRV + MVC + RTV* | 7 | 0.29 | 1854 | 76.64 |
| *2. POST-CP* | *DRV + MVC + RTV + AZT* | 1 | 0.04 | 1855 | 76.68 |
| *2. POST-CP* | *DRV + MVC + RTV + TDF/FTC* | 1 | 0.04 | 1856 | 76.73 |
| *2. POST-CP* | *DRV + NVP + RTV* | 1 | 0.04 | 1857 | 76.77 |
| *2. POST-CP* | *DRV + RAL + RTV* | 26 | 1.07 | 1883 | 77.84 |
| *2. POST-CP* | *DRV + RAL + RTV + NVP* | 1 | 0.04 | 1884 | 77.88 |
| *2. POST-CP* | *DRV + RAL + RTV + TDF* | 4 | 0.17 | 1888 | 78.05 |
| *2. POST-CP* | *DRV + RAL + RTV + TDF/FTC* | 6 | 0.25 | 1894 | 78.30 |
| *2. POST-CP* | *DRV + RTV* | 41 | 1.69 | 1935 | 79.99 |
| *2. POST-CP* | *DRV + RTV + FTC* | 2 | 0.08 | 1937 | 80.07 |
| *2. POST-CP* | *DRV + RTV + TDF* | 1 | 0.04 | 1938 | 80.12 |
| *2. POST-CP* | *DRV + RTV + TDF + MVC* | 1 | 0.04 | 1939 | 80.16 |
| *2. POST-CP* | *DRV + RTV + TDF/FTC* | 118 | 4.88 | 2057 | 85.04 |
| *2. POST-CP* | *DRV + RTV + TDF/FTC + NVP* | 1 | 0.04 | 2058 | 85.08 |
| *2. POST-CP* | *DRV + TDF/FTC* | 11 | 0.45 | 2069 | 85.53 |
| *2. POST-CP* | *EFV + TDF/FTC* | 50 | 2.07 | 2119 | 87.60 |
| *2. POST-CP* | *ETV + LPV/r* | 1 | 0.04 | 2120 | 87.64 |
| *2. POST-CP* | *ETV + RAL + TDF/FTC* | 1 | 0.04 | 2121 | 87.68 |
| *2. POST-CP* | *ETV + TDF/FTC* | 9 | 0.37 | 2130 | 88.05 |
| *2. POST-CP* | *FPV + NVP + FTC* | 1 | 0.04 | 2131 | 88.09 |
| *2. POST-CP* | *FPV + RTV* | 1 | 0.04 | 2132 | 88.14 |
| *2. POST-CP* | *FPV + RTV + RAL* | 1 | 0.04 | 2133 | 88.18 |
| *2. POST-CP* | *FPV + RTV + TDF/FTC* | 6 | 0.25 | 2139 | 88.42 |
| *2. POST-CP* | *FPV + TDF/FTC* | 6 | 0.25 | 2145 | 88.67 |
| *2. POST-CP* | *LPV/r* | 20 | 0.83 | 2165 | 89.50 |
| *2. POST-CP* | *LPV/r + MCV* | 1 | 0.04 | 2166 | 89.54 |
| *2. POST-CP* | *LPV/r + RAL* | 8 | 0.33 | 2174 | 89.87 |
| *2. POST-CP* | *LPV/r + RAL + 3TC* | 1 | 0.04 | 2175 | 89.91 |
| *2. POST-CP* | *LPV/r + TDF/FTC* | 24 | 0.99 | 2199 | 90.91 |
| *2. POST-CP* | *LPV/r +MVC* | 2 | 0.08 | 2201 | 90.99 |
| *2. POST-CP* | *MVC + DRV + RAL + RTV* | 1 | 0.04 | 2202 | 91.03 |
| *2. POST-CP* | *MVC + RAL* | 6 | 0.25 | 2208 | 91.28 |
| *2. POST-CP* | *MVC + RAL + TDF* | 1 | 0.04 | 2209 | 91.32 |
| *2. POST-CP* | *MVC + RAL + TDF/FTC* | 1 | 0.04 | 2210 | 91.36 |
| *2. POST-CP* | *MVC + TDF/FTC* | 1 | 0.04 | 2211 | 91.40 |
| *2. POST-CP* | *NVP + ATV* | 1 | 0.04 | 2212 | 91.44 |
| *2. POST-CP* | *NVP + RAL* | 1 | 0.04 | 2213 | 91.48 |
| *2. POST-CP* | *NVP + TDF/FTC* | 66 | 2.73 | 2279 | 94.21 |
| *2. POST-CP* | *NVP+ LPV/r* | 1 | 0.04 | 2280 | 94.25 |
| *2. POST-CP* | *RAL + TDF + FTC* | 1 | 0.04 | 2281 | 94.30 |
| *2. POST-CP* | *RAL + TDF/FTC* | 23 | 0.95 | 2304 | 95.25 |
| *2. POST-CP* | *RAL + TDF/FTC + NVP* | 1 | 0.04 | 2305 | 95.29 |
| *2. POST-CP* | *TDF + ATV + RTV + ABC* | 1 | 0.04 | 2306 | 95.33 |
| *2. POST-CP* | *TDF/FTC* | 3 | 0.12 | 2309 | 95.45 |
| *2. POST-CP* | *TDF/FTC/EFV* | 108 | 4.46 | 2417 | 99.92 |
| *2. POST-CP* | *ddl + RTV + ATV + 3TC* | 1 | 0.04 | 2418 | 99.96 |
| *2. POST-CP* | *drug holiday* | 1 | 0.04 | 2419 | 100.00 |
