# Supplementary material for: HIV Clinical Pathway: A New Approach to Combine Guidelines and Sustainability of Anti-Retroviral Treatment in Italy
Source: PLoS One. 2016 Dec 28;11(12):e0168399. doi: 10.1371/journal.pone.0168399 (PMC5193418; doi:10.1371/journal.pone.0168399)
Supplement: S2 Table — first line therapy. (DOCX) [file pone.0168399.s002.docx]

**S2 Table. HAART regimen by CP category: *category=1. first line therapy***

| *PERIOD* | *HAART* | *Frequency* | *Percent* | *Cumulative Frequency* | *Cumulative Percent* |
| --- | --- | --- | --- | --- | --- |
| *1. PRE-CP* | *3TC + RAL + TDF* | 1 | 0.17 | 1 | 0.17 |
| *1. PRE-CP* | *3TC/ABC + ATV* | 1 | 0.17 | 2 | 0.34 |
| *1. PRE-CP* | *3TC/ABC + ATV + RTV* | 6 | 1.01 | 8 | 1.35 |
| *1. PRE-CP* | *3TC/ABC + DRV + RTV* | 2 | 0.34 | 10 | 1.69 |
| *1. PRE-CP* | *3TC/ABC + EFV* | 3 | 0.51 | 13 | 2.20 |
| *1. PRE-CP* | *3TC/ABC + LPV/r* | 2 | 0.34 | 15 | 2.53 |
| *1. PRE-CP* | *3TC/ABC + NVP* | 4 | 0.68 | 19 | 3.21 |
| *1. PRE-CP* | *3TC/ABC + RAL* | 1 | 0.17 | 20 | 3.38 |
| *1. PRE-CP* | *3TC/ABC/AZT* | 1 | 0.17 | 21 | 3.55 |
| *1. PRE-CP* | *3TC/AZT + ATV + RTV* | 3 | 0.51 | 24 | 4.05 |
| *1. PRE-CP* | *3TC/AZT + DRV + RTV* | 1 | 0.17 | 25 | 4.22 |
| *1. PRE-CP* | *3TC/AZT + EFV* | 3 | 0.51 | 28 | 4.73 |
| *1. PRE-CP* | *3TC/AZT + LPV/r* | 5 | 0.84 | 33 | 5.57 |
| *1. PRE-CP* | *3TC/AZT + LPV/r + MVC* | 1 | 0.17 | 34 | 5.74 |
| *1. PRE-CP* | *3TC/AZT + NVP* | 2 | 0.34 | 36 | 6.08 |
| *1. PRE-CP* | *ATV + MVC + RAL* | 1 | 0.17 | 37 | 6.25 |
| *1. PRE-CP* | *ATV + MVC + RAL + RTV* | 1 | 0.17 | 38 | 6.42 |
| *1. PRE-CP* | *ATV + RTV* | 1 | 0.17 | 39 | 6.59 |
| *1. PRE-CP* | *ATV + RTV + TDF/FTC* | 61 | 10.30 | 100 | 16.89 |
| *1. PRE-CP* | *ATV + TDF/FTC* | 2 | 0.34 | 102 | 17.23 |
| *1. PRE-CP* | *DRV + ETV + MVC + RTV* | 1 | 0.17 | 103 | 17.40 |
| *1. PRE-CP* | *DRV + MVC + RAL + RTV* | 5 | 0.84 | 108 | 18.24 |
| *1. PRE-CP* | *DRV + RAL + RTV + TDF/FTC* | 1 | 0.17 | 109 | 18.41 |
| *1. PRE-CP* | *DRV + RTV + TDF/FTC* | 77 | 13.01 | 186 | 31.42 |
| *1. PRE-CP* | *DRV + TDF/FTC* | 1 | 0.17 | 187 | 31.59 |
| *1. PRE-CP* | *EFV + TDF/FTC* | 27 | 4.56 | 214 | 36.15 |
| *1. PRE-CP* | *FPV + RTV + TDF/FTC* | 2 | 0.34 | 216 | 36.49 |
| *1. PRE-CP* | *LPV/r + TDF/FTC* | 23 | 3.89 | 239 | 40.37 |
| *1. PRE-CP* | *NVP + TDF/FTC* | 8 | 1.35 | 247 | 41.72 |
| *1. PRE-CP* | *RAL + TDF/FTC* | 7 | 1.18 | 254 | 42.91 |
| *1. PRE-CP* | *TDF/FTC/EFV* | 70 | 11.82 | 324 | 54.73 |
| *2. POST-CP* | *3TC + DRV + RTV* | 1 | 0.17 | 325 | 54.90 |
| *2. POST-CP* | *3TC + NVP + TDF* | 1 | 0.17 | 326 | 55.07 |
| *2. POST-CP* | *3TC/ABC + ATV + RTV* | 13 | 2.20 | 339 | 57.26 |
| *2. POST-CP* | *3TC/ABC + DRV + RTV* | 4 | 0.68 | 343 | 57.94 |
| *2. POST-CP* | *3TC/ABC + EFV* | 3 | 0.51 | 346 | 58.45 |
| *2. POST-CP* | *3TC/ABC + LPV/r* | 1 | 0.17 | 347 | 58.61 |
| *2. POST-CP* | *3TC/ABC + NVP* | 4 | 0.68 | 351 | 59.29 |
| *2. POST-CP* | *3TC/AZT + ATV + RTV* | 1 | 0.17 | 352 | 59.46 |
| *2. POST-CP* | *3TC/AZT + DRV + RTV* | 1 | 0.17 | 353 | 59.63 |
| *2. POST-CP* | *3TC/AZT + EFV* | 2 | 0.34 | 355 | 59.97 |
| *2. POST-CP* | *3TC/AZT + LPV/r* | 10 | 1.69 | 365 | 61.66 |
| *2. POST-CP* | *ATV + RTV + TDF/FTC* | 58 | 9.80 | 423 | 71.45 |
| *2. POST-CP* | *DRV + ETV + RTV* | 2 | 0.34 | 425 | 71.79 |
| *2. POST-CP* | *DRV + MVC + RTV* | 1 | 0.17 | 426 | 71.96 |
| *2. POST-CP* | *DRV + RAL + RTV* | 2 | 0.34 | 428 | 72.30 |
| *2. POST-CP* | *DRV + RAL + RTV + TDF/FTC* | 2 | 0.34 | 430 | 72.64 |
| *2. POST-CP* | *DRV + RTV + TDF/FTC* | 52 | 8.78 | 482 | 81.42 |
| *2. POST-CP* | *DRV + TDF/FTC* | 8 | 1.35 | 490 | 82.77 |
| *2. POST-CP* | *EFV + TDF/FTC* | 33 | 5.57 | 523 | 88.34 |
| *2. POST-CP* | *LPV/r + RAL* | 1 | 0.17 | 524 | 88.51 |
| *2. POST-CP* | *LPV/r + TDF/FTC* | 8 | 1.35 | 532 | 89.86 |
| *2. POST-CP* | *LPV/r +MVC* | 1 | 0.17 | 533 | 90.03 |
| *2. POST-CP* | *NVP + TDF/FTC* | 8 | 1.35 | 541 | 91.39 |
| *2. POST-CP* | *RAL + TDF/FTC* | 3 | 0.51 | 544 | 91.89 |
| *2. POST-CP* | *TDF/FTC* | 1 | 0.17 | 545 | 92.06 |
| *2. POST-CP* | *TDF/FTC/EFV* | 47 | 7.94 | 592 | 100.00 |
