# Supplementary material for: HIV Clinical Pathway: A New Approach to Combine Guidelines and Sustainability of Anti-Retroviral Treatment in Italy
Source: PLoS One. 2016 Dec 28;11(12):e0168399. doi: 10.1371/journal.pone.0168399 (PMC5193418; doi:10.1371/journal.pone.0168399)
Supplement: S3 Table — Toxicity/other. (DOCX) [file pone.0168399.s003.docx]

**S3 Table. HAART regimen by CP category: category=2. Toxicity/other**

| *PERIOD* | *HAART* | *Frequency* | *Percent* | *Cumulative Frequency* | *Cumulative Percent* |
| --- | --- | --- | --- | --- | --- |
| *1. PRE-CP* | *3TC + ABC + ATV + RTV* | 4 | 0.28 | 4 | 0.28 |
| *1. PRE-CP* | *3TC + ATV* | 3 | 0.21 | 7 | 0.48 |
| *1. PRE-CP* | *3TC + ATV + RAL* | 4 | 0.28 | 11 | 0.76 |
| *1. PRE-CP* | *3TC + ATV + RAL + RTV* | 3 | 0.21 | 14 | 0.97 |
| *1. PRE-CP* | *3TC + ATV + RTV* | 5 | 0.34 | 19 | 1.31 |
| *1. PRE-CP* | *3TC + ATV + RTV + TDF* | 3 | 0.21 | 22 | 1.52 |
| *1. PRE-CP* | *3TC + ATV + TDF* | 1 | 0.07 | 23 | 1.59 |
| *1. PRE-CP* | *3TC + ATV + ddl* | 3 | 0.21 | 26 | 1.79 |
| *1. PRE-CP* | *3TC + DRV + ATV + RTV* | 1 | 0.07 | 27 | 1.86 |
| *1. PRE-CP* | *3TC + DRV + EFV + RAL + RTV* | 1 | 0.07 | 28 | 1.93 |
| *1. PRE-CP* | *3TC + DRV + EFV + RTV* | 1 | 0.07 | 29 | 2.00 |
| *1. PRE-CP* | *3TC + DRV + ETV + RTV* | 2 | 0.14 | 31 | 2.14 |
| *1. PRE-CP* | *3TC + DRV + RAL + RTV* | 2 | 0.14 | 33 | 2.28 |
| *1. PRE-CP* | *3TC + DRV + RTV* | 1 | 0.07 | 34 | 2.34 |
| *1. PRE-CP* | *3TC + DRV + RTV + TDF* | 1 | 0.07 | 35 | 2.41 |
| *1. PRE-CP* | *3TC + EFV+ RAL* | 1 | 0.07 | 36 | 2.48 |
| *1. PRE-CP* | *3TC + FPV* | 1 | 0.07 | 37 | 2.55 |
| *1. PRE-CP* | *3TC + FPV + RTV + TDF* | 1 | 0.07 | 38 | 2.62 |
| *1. PRE-CP* | *3TC + LPV/r* | 3 | 0.21 | 41 | 2.83 |
| *1. PRE-CP* | *3TC + NVP + RAL* | 2 | 0.14 | 43 | 2.97 |
| *1. PRE-CP* | *3TC + RAL* | 3 | 0.21 | 46 | 3.17 |
| *1. PRE-CP* | *3TC + RAL + NVP + MVC* | 1 | 0.07 | 47 | 3.24 |
| *1. PRE-CP* | *3TC + RTV + ATV + RAL* | 1 | 0.07 | 48 | 3.31 |
| *1. PRE-CP* | *3TC + TDF* | 1 | 0.07 | 49 | 3.38 |
| *1. PRE-CP* | *3TC + ddl + DRV + RTV* | 1 | 0.07 | 50 | 3.45 |
| *1. PRE-CP* | *3TC + ddl + MVC* | 1 | 0.07 | 51 | 3.52 |
| *1. PRE-CP* | *3TC + ddl + NVP* | 1 | 0.07 | 52 | 3.59 |
| *1. PRE-CP* | *3TC/ABC* | 2 | 0.14 | 54 | 3.72 |
| *1. PRE-CP* | *3TC/ABC + ATV* | 34 | 2.34 | 88 | 6.07 |
| *1. PRE-CP* | *3TC/ABC + ATV + NVP* | 1 | 0.07 | 89 | 6.14 |
| *1. PRE-CP* | *3TC/ABC + ATV + RAL + RTV* | 1 | 0.07 | 90 | 6.21 |
| *1. PRE-CP* | *3TC/ABC + ATV + RTV* | 21 | 1.45 | 111 | 7.66 |
| *1. PRE-CP* | *3TC/ABC + DRV + RAL + RTV* | 1 | 0.07 | 112 | 7.72 |
| *1. PRE-CP* | *3TC/ABC + DRV + RTV* | 9 | 0.62 | 121 | 8.34 |
| *1. PRE-CP* | *3TC/ABC + EFV* | 10 | 0.69 | 131 | 9.03 |
| *1. PRE-CP* | *3TC/ABC + ETV* | 1 | 0.07 | 132 | 9.10 |
| *1. PRE-CP* | *3TC/ABC + ETV + RAL* | 1 | 0.07 | 133 | 9.17 |
| *1. PRE-CP* | *3TC/ABC + FPV + RTV* | 1 | 0.07 | 134 | 9.24 |
| *1. PRE-CP* | *3TC/ABC + LPV/r* | 10 | 0.69 | 144 | 9.93 |
| *1. PRE-CP* | *3TC/ABC + NVP* | 15 | 1.03 | 159 | 10.97 |
| *1. PRE-CP* | *3TC/ABC + RAL* | 3 | 0.21 | 162 | 11.17 |
| *1. PRE-CP* | *3TC/ABC/AZT* | 3 | 0.21 | 165 | 11.38 |
| *1. PRE-CP* | *3TC/AZT* | 3 | 0.21 | 168 | 11.59 |
| *1. PRE-CP* | *3TC/AZT + ATV* | 3 | 0.21 | 171 | 11.79 |
| *1. PRE-CP* | *3TC/AZT + ATV + RTV* | 6 | 0.41 | 177 | 12.21 |
| *1. PRE-CP* | *3TC/AZT + DRV + RAL + RTV* | 1 | 0.07 | 178 | 12.28 |
| *1. PRE-CP* | *3TC/AZT + DRV + RTV* | 2 | 0.14 | 180 | 12.41 |
| *1. PRE-CP* | *3TC/AZT + EFV* | 1 | 0.07 | 181 | 12.48 |
| *1. PRE-CP* | *3TC/AZT + FPV + RTV* | 1 | 0.07 | 182 | 12.55 |
| *1. PRE-CP* | *3TC/AZT + LPV/r* | 5 | 0.34 | 187 | 12.90 |
| *1. PRE-CP* | *3TC/AZT + NVP* | 6 | 0.41 | 193 | 13.31 |
| *1. PRE-CP* | *3TC/AZT + RAL* | 3 | 0.21 | 196 | 13.52 |
| *1. PRE-CP* | *3TC/AZT + RTV + SQV* | 1 | 0.07 | 197 | 13.59 |
| *1. PRE-CP* | *ATV* | 8 | 0.55 | 205 | 14.14 |
| *1. PRE-CP* | *ATV + FTC + RAL* | 2 | 0.14 | 207 | 14.28 |
| *1. PRE-CP* | *ATV + FTC + RAL + RTV* | 1 | 0.07 | 208 | 14.34 |
| *1. PRE-CP* | *ATV + MVC + RTV + TDF/FTC* | 2 | 0.14 | 210 | 14.48 |
| *1. PRE-CP* | *ATV + RAL* | 21 | 1.45 | 231 | 15.93 |
| *1. PRE-CP* | *ATV + RAL + RTV + TDF* | 1 | 0.07 | 232 | 16.00 |
| *1. PRE-CP* | *ATV + RAL + TDF/FTC* | 1 | 0.07 | 233 | 16.07 |
| *1. PRE-CP* | *ATV + RTV* | 21 | 1.45 | 254 | 17.52 |
| *1. PRE-CP* | *ATV + RTV + FVP* | 1 | 0.07 | 255 | 17.59 |
| *1. PRE-CP* | *ATV + RTV + TDF/FTC* | 91 | 6.28 | 346 | 23.86 |
| *1. PRE-CP* | *ATV + TDF/FTC* | 25 | 1.72 | 371 | 25.59 |
| *1. PRE-CP* | *ATV + ddl* | 1 | 0.07 | 372 | 25.66 |
| *1. PRE-CP* | *ATV + ddl + RTV + TDF* | 1 | 0.07 | 373 | 25.72 |
| *1. PRE-CP* | *ATV+SQV* | 1 | 0.07 | 374 | 25.79 |
| *1. PRE-CP* | *DRV + ETV + RAL + RTV* | 1 | 0.07 | 375 | 25.86 |
| *1. PRE-CP* | *DRV + ETV + RAL + RTV + TDF/FTC* | 1 | 0.07 | 376 | 25.93 |
| *1. PRE-CP* | *DRV + ETV + RTV* | 1 | 0.07 | 377 | 26.00 |
| *1. PRE-CP* | *DRV + MVC + RTV* | 4 | 0.28 | 381 | 26.28 |
| *1. PRE-CP* | *DRV + RAL + RTV* | 10 | 0.69 | 391 | 26.97 |
| *1. PRE-CP* | *DRV + RAL + RTV + TDF* | 1 | 0.07 | 392 | 27.03 |
| *1. PRE-CP* | *DRV + RAL + RTV + TDF/FTC* | 3 | 0.21 | 395 | 27.24 |
| *1. PRE-CP* | *DRV + RTV* | 11 | 0.76 | 406 | 28.00 |
| *1. PRE-CP* | *DRV + RTV + TDF* | 1 | 0.07 | 407 | 28.07 |
| *1. PRE-CP* | *DRV + RTV + TDF/FTC* | 25 | 1.72 | 432 | 29.79 |
| *1. PRE-CP* | *DRV + TDF/FTC* | 1 | 0.07 | 433 | 29.86 |
| *1. PRE-CP* | *EFV* | 1 | 0.07 | 434 | 29.93 |
| *1. PRE-CP* | *EFV + RAL* | 1 | 0.07 | 435 | 30.00 |
| *1. PRE-CP* | *EFV + TDF/FTC* | 14 | 0.97 | 449 | 30.97 |
| *1. PRE-CP* | *ETV + MVC + RAL* | 1 | 0.07 | 450 | 31.03 |
| *1. PRE-CP* | *ETV + RAL* | 4 | 0.28 | 454 | 31.31 |
| *1. PRE-CP* | *ETV + RAL + TDF/FTC* | 1 | 0.07 | 455 | 31.38 |
| *1. PRE-CP* | *ETV + TDF/FTC* | 1 | 0.07 | 456 | 31.45 |
| *1. PRE-CP* | *FPV + RAL + TDF/FTC* | 1 | 0.07 | 457 | 31.52 |
| *1. PRE-CP* | *FPV + RTV* | 3 | 0.21 | 460 | 31.72 |
| *1. PRE-CP* | *FPV + RTV + TDF/FTC* | 10 | 0.69 | 470 | 32.41 |
| *1. PRE-CP* | *FPV + TDF/FTC* | 5 | 0.34 | 475 | 32.76 |
| *1. PRE-CP* | *FTC + MVC + RAL* | 1 | 0.07 | 476 | 32.83 |
| *1. PRE-CP* | *LPV/r* | 24 | 1.66 | 500 | 34.48 |
| *1. PRE-CP* | *LPV/r + RAL* | 2 | 0.14 | 502 | 34.62 |
| *1. PRE-CP* | *LPV/r + TDF/FTC* | 24 | 1.66 | 526 | 36.28 |
| *1. PRE-CP* | *MVC + RAL* | 1 | 0.07 | 527 | 36.34 |
| *1. PRE-CP* | *MVC + RAL + T20* | 1 | 0.07 | 528 | 36.41 |
| *1. PRE-CP* | *NVP + ATV* | 1 | 0.07 | 529 | 36.48 |
| *1. PRE-CP* | *NVP + RAL* | 1 | 0.07 | 530 | 36.55 |
| *1. PRE-CP* | *NVP + TDF/FTC* | 46 | 3.17 | 576 | 39.72 |
| *1. PRE-CP* | *RAL + T20* | 1 | 0.07 | 577 | 39.79 |
| *1. PRE-CP* | *RAL + TDF/FTC* | 26 | 1.79 | 603 | 41.59 |
| *1. PRE-CP* | *T20 + TDF/FTC* | 1 | 0.07 | 604 | 41.66 |
| *1. PRE-CP* | *TDF + ATV + RTV + AZT* | 1 | 0.07 | 605 | 41.72 |
| *1. PRE-CP* | *TDF/FTC* | 6 | 0.41 | 611 | 42.14 |
| *1. PRE-CP* | *TDF/FTC + NVP+ LPV/r* | 1 | 0.07 | 612 | 42.21 |
| *1. PRE-CP* | *TDF/FTC/EFV* | 114 | 7.86 | 726 | 50.07 |
| *1. PRE-CP* | *TPV + RTV + MVC + RAL* | 1 | 0.07 | 727 | 50.14 |
| *1. PRE-CP* | *ddl + DRV + FTC + RTV* | 1 | 0.07 | 728 | 50.21 |
| *1. PRE-CP* | *ddl + DRV + RAL + RTV* | 2 | 0.14 | 730 | 50.34 |
| *1. PRE-CP* | *ddl + FTC* | 1 | 0.07 | 731 | 50.41 |
| *1. PRE-CP* | *sospensione* | 2 | 0.14 | 733 | 50.55 |
| *2. POST-CP* | *3TC* | 2 | 0.14 | 735 | 50.69 |
| *2. POST-CP* | *3TC + DRV + RTV* | 1 | 0.07 | 736 | 50.76 |
| *2. POST-CP* | *3TC + ABC + ATV + RTV* | 1 | 0.07 | 737 | 50.83 |
| *2. POST-CP* | *3TC + ATV* | 10 | 0.69 | 747 | 51.52 |
| *2. POST-CP* | *3TC + ATV + RAL* | 2 | 0.14 | 749 | 51.66 |
| *2. POST-CP* | *3TC + ATV + RTV* | 4 | 0.28 | 753 | 51.93 |
| *2. POST-CP* | *3TC + ATV + RTV + TDF* | 1 | 0.07 | 754 | 52.00 |
| *2. POST-CP* | *3TC + ATV + TDF/FTC* | 1 | 0.07 | 755 | 52.07 |
| *2. POST-CP* | *3TC + ATV+ DRV* | 1 | 0.07 | 756 | 52.14 |
| *2. POST-CP* | *3TC + AZT + RAL* | 1 | 0.07 | 757 | 52.21 |
| *2. POST-CP* | *3TC + DRV + NVP + RTV* | 1 | 0.07 | 758 | 52.28 |
| *2. POST-CP* | *3TC + DRV + RTV* | 5 | 0.34 | 763 | 52.62 |
| *2. POST-CP* | *3TC + DRV + RTV + TDF* | 1 | 0.07 | 764 | 52.69 |
| *2. POST-CP* | *3TC + EFV* | 1 | 0.07 | 765 | 52.76 |
| *2. POST-CP* | *3TC + FPV +RAL* | 1 | 0.07 | 766 | 52.83 |
| *2. POST-CP* | *3TC + LPV/r* | 5 | 0.34 | 771 | 53.17 |
| *2. POST-CP* | *3TC + LPV/r + TDF* | 2 | 0.14 | 773 | 53.31 |
| *2. POST-CP* | *3TC + MVC + LVP/r* | 1 | 0.07 | 774 | 53.38 |
| *2. POST-CP* | *3TC + MVC + RAL* | 1 | 0.07 | 775 | 53.45 |
| *2. POST-CP* | *3TC + NVP + RAL* | 1 | 0.07 | 776 | 53.52 |
| *2. POST-CP* | *3TC + NVP + TDF* | 1 | 0.07 | 777 | 53.59 |
| *2. POST-CP* | *3TC + ddl + DRV + RTV* | 1 | 0.07 | 778 | 53.66 |
| *2. POST-CP* | *3TC + ddl + EFV* | 1 | 0.07 | 779 | 53.72 |
| *2. POST-CP* | *3TC + ddl + NVP* | 1 | 0.07 | 780 | 53.79 |
| *2. POST-CP* | *3TC/ABC* | 8 | 0.55 | 788 | 54.34 |
| *2. POST-CP* | *3TC/ABC + ATV* | 14 | 0.97 | 802 | 55.31 |
| *2. POST-CP* | *3TC/ABC + ATV + RTV* | 42 | 2.90 | 844 | 58.21 |
| *2. POST-CP* | *3TC/ABC + AZT* | 1 | 0.07 | 845 | 58.28 |
| *2. POST-CP* | *3TC/ABC + DRV + RAL + RTV* | 1 | 0.07 | 846 | 58.34 |
| *2. POST-CP* | *3TC/ABC + DRV + RTV* | 17 | 1.17 | 863 | 59.52 |
| *2. POST-CP* | *3TC/ABC + DRV + RTV + TDF* | 1 | 0.07 | 864 | 59.59 |
| *2. POST-CP* | *3TC/ABC + EFV* | 15 | 1.03 | 879 | 60.62 |
| *2. POST-CP* | *3TC/ABC + ETV* | 7 | 0.48 | 886 | 61.10 |
| *2. POST-CP* | *3TC/ABC + FPV + RTV* | 3 | 0.21 | 889 | 61.31 |
| *2. POST-CP* | *3TC/ABC + LPV/r* | 11 | 0.76 | 900 | 62.07 |
| *2. POST-CP* | *3TC/ABC + NVP* | 47 | 3.24 | 947 | 65.31 |
| *2. POST-CP* | *3TC/ABC + NVP + RAL* | 2 | 0.14 | 949 | 65.45 |
| *2. POST-CP* | *3TC/ABC + RAL* | 9 | 0.62 | 958 | 66.07 |
| *2. POST-CP* | *3TC/ABC + SQV + RTV* | 2 | 0.14 | 960 | 66.21 |
| *2. POST-CP* | *3TC/ABC/AZT* | 2 | 0.14 | 962 | 66.34 |
| *2. POST-CP* | *3TC/AZT + ATV + RTV* | 7 | 0.48 | 969 | 66.83 |
| *2. POST-CP* | *3TC/AZT + EFV* | 2 | 0.14 | 971 | 66.97 |
| *2. POST-CP* | *3TC/AZT + LPV/r* | 2 | 0.14 | 973 | 67.10 |
| *2. POST-CP* | *3TC/AZT + RAL* | 3 | 0.21 | 976 | 67.31 |
| *2. POST-CP* | *ABC + TDF+ ATV + RTV* | 1 | 0.07 | 977 | 67.38 |
| *2. POST-CP* | *ATV* | 6 | 0.41 | 983 | 67.79 |
| *2. POST-CP* | *ATV + TDF/FTC* | 1 | 0.07 | 984 | 67.86 |
| *2. POST-CP* | *ATV + DRV + RTV + TDF/FTC* | 1 | 0.07 | 985 | 67.93 |
| *2. POST-CP* | *ATV + ETV + RAL + RTV* | 1 | 0.07 | 986 | 68.00 |
| *2. POST-CP* | *ATV + FTC* | 1 | 0.07 | 987 | 68.07 |
| *2. POST-CP* | *ATV + FTC + RTV* | 1 | 0.07 | 988 | 68.14 |
| *2. POST-CP* | *ATV + RAL + RTV* | 5 | 0.34 | 993 | 68.48 |
| *2. POST-CP* | *ATV + RAL + RTV + TDF* | 1 | 0.07 | 994 | 68.55 |
| *2. POST-CP* | *ATV + RTV* | 20 | 1.38 | 1014 | 69.93 |
| *2. POST-CP* | *ATV + RTV + FTC + MVC* | 1 | 0.07 | 1015 | 70.00 |
| *2. POST-CP* | *ATV + RTV + TDF/FTC* | 84 | 5.79 | 1099 | 75.79 |
| *2. POST-CP* | *ATV + TDF/FTC* | 10 | 0.69 | 1109 | 76.48 |
| *2. POST-CP* | *ATV+FTC* | 2 | 0.14 | 1111 | 76.62 |
| *2. POST-CP* | *AZT + TDF/FTC* | 1 | 0.07 | 1112 | 76.69 |
| *2. POST-CP* | *DRV + ETV + RAL + RTV* | 3 | 0.21 | 1115 | 76.90 |
| *2. POST-CP* | *DRV + ETV + RTV* | 7 | 0.48 | 1122 | 77.38 |
| *2. POST-CP* | *DRV + MVC + RTV* | 2 | 0.14 | 1124 | 77.52 |
| *2. POST-CP* | *DRV + NVP + RTV* | 1 | 0.07 | 1125 | 77.59 |
| *2. POST-CP* | *DRV + RAL + RTV* | 11 | 0.76 | 1136 | 78.34 |
| *2. POST-CP* | *DRV + RAL + RTV + NVP* | 1 | 0.07 | 1137 | 78.41 |
| *2. POST-CP* | *DRV + RAL + RTV + TDF* | 2 | 0.14 | 1139 | 78.55 |
| *2. POST-CP* | *DRV + RAL + RTV + TDF/FTC* | 2 | 0.14 | 1141 | 78.69 |
| *2. POST-CP* | *DRV + RTV* | 39 | 2.69 | 1180 | 81.38 |
| *2. POST-CP* | *DRV + RTV + FTC* | 2 | 0.14 | 1182 | 81.52 |
| *2. POST-CP* | *DRV + RTV + TDF/FTC* | 49 | 3.38 | 1231 | 84.90 |
| *2. POST-CP* | *DRV + RTV + TDF/FTC + NVP* | 1 | 0.07 | 1232 | 84.97 |
| *2. POST-CP* | *DRV + TDF/FTC* | 2 | 0.14 | 1234 | 85.10 |
| *2. POST-CP* | *EFV + TDF/FTC* | 17 | 1.17 | 1251 | 86.28 |
| *2. POST-CP* | *ETV + LPV/r* | 1 | 0.07 | 1252 | 86.34 |
| *2. POST-CP* | *ETV + RAL + TDF/FTC* | 1 | 0.07 | 1253 | 86.41 |
| *2. POST-CP* | *ETV + TDF/FTC* | 5 | 0.34 | 1258 | 86.76 |
| *2. POST-CP* | *FPV + NVP + FTC* | 1 | 0.07 | 1259 | 86.83 |
| *2. POST-CP* | *FPV + RTV* | 1 | 0.07 | 1260 | 86.90 |
| *2. POST-CP* | *FPV + RTV + RAL* | 1 | 0.07 | 1261 | 86.97 |
| *2. POST-CP* | *FPV + RTV + TDF/FTC* | 5 | 0.34 | 1266 | 87.31 |
| *2. POST-CP* | *FPV + TDF/FTC* | 5 | 0.34 | 1271 | 87.66 |
| *2. POST-CP* | *LPV/r* | 20 | 1.38 | 1291 | 89.03 |
| *2. POST-CP* | *LPV/r + MCV* | 1 | 0.07 | 1292 | 89.10 |
| *2. POST-CP* | *LPV/r + RAL* | 5 | 0.34 | 1297 | 89.45 |
| *2. POST-CP* | *LPV/r + TDF/FTC* | 7 | 0.48 | 1304 | 89.93 |
| *2. POST-CP* | *LPV/r +MVC* | 1 | 0.07 | 1305 | 90.00 |
| *2. POST-CP* | *MVC + RAL* | 6 | 0.41 | 1311 | 90.41 |
| *2. POST-CP* | *MVC + RAL + TDF/FTC* | 1 | 0.07 | 1312 | 90.48 |
| *2. POST-CP* | *MVC + TDF/FTC* | 1 | 0.07 | 1313 | 90.55 |
| *2. POST-CP* | *NVP + ATV* | 1 | 0.07 | 1314 | 90.62 |
| *2. POST-CP* | *NVP + RAL* | 1 | 0.07 | 1315 | 90.69 |
| *2. POST-CP* | *NVP + TDF/FTC* | 56 | 3.86 | 1371 | 94.55 |
| *2. POST-CP* | *NVP+ LPV/r* | 1 | 0.07 | 1372 | 94.62 |
| *2. POST-CP* | *RAL + TDF + FTC* | 1 | 0.07 | 1373 | 94.69 |
| *2. POST-CP* | *RAL + TDF/FTC* | 15 | 1.03 | 1388 | 95.72 |
| *2. POST-CP* | *RAL + TDF/FTC + NVP* | 1 | 0.07 | 1389 | 95.79 |
| *2. POST-CP* | *TDF/FTC* | 2 | 0.14 | 1391 | 95.93 |
| *2. POST-CP* | *TDF/FTC/EFV* | 57 | 3.93 | 1448 | 99.86 |
| *2. POST-CP* | *ddl + RTV + ATV + 3TC* | 1 | 0.07 | 1449 | 99.93 |
| *2. POST-CP* | *drug holiday* | 1 | 0.07 | 1450 | 100.00 |
