# Supplementary material for: HIV Clinical Pathway: A New Approach to Combine Guidelines and Sustainability of Anti-Retroviral Treatment in Italy
Source: PLoS One. 2016 Dec 28;11(12):e0168399. doi: 10.1371/journal.pone.0168399 (PMC5193418; doi:10.1371/journal.pone.0168399)
Supplement: S4 Table — Failure. (DOCX) [file pone.0168399.s004.docx]

**S4 Table. HAART regimen by CP category: category=3. Failure**

| *PERIOD* | *HAART* | *Frequency* | *Percent* | *Cumulative Frequency* | *Cumulative Percent* |
| --- | --- | --- | --- | --- | --- |
| *1. PRE-CP* | *3TC* | 4 | 1.06 | 4 | 1.06 |
| *1. PRE-CP* | *3TC + ATV* | 1 | 0.27 | 5 | 1.33 |
| *1. PRE-CP* | *3TC + ATV + MVC* | 1 | 0.27 | 6 | 1.59 |
| *1. PRE-CP* | *3TC + ATV + RTV + TDF* | 1 | 0.27 | 7 | 1.86 |
| *1. PRE-CP* | *3TC + AZT + DRV + RTV* | 1 | 0.27 | 8 | 2.12 |
| *1. PRE-CP* | *3TC + AZT + RAL* | 1 | 0.27 | 9 | 2.39 |
| *1. PRE-CP* | *3TC + DRV + ATV + RTV* | 1 | 0.27 | 10 | 2.65 |
| *1. PRE-CP* | *3TC + DRV + ETV + RTV* | 1 | 0.27 | 11 | 2.92 |
| *1. PRE-CP* | *3TC + DRV + RAL + RTV* | 1 | 0.27 | 12 | 3.18 |
| *1. PRE-CP* | *3TC + DRV + RTV* | 2 | 0.53 | 14 | 3.71 |
| *1. PRE-CP* | *3TC + LPV/r + TDF* | 1 | 0.27 | 15 | 3.98 |
| *1. PRE-CP* | *3TC + STV + DRV + ETV + MVC + RTV + T20* | 1 | 0.27 | 16 | 4.24 |
| *1. PRE-CP* | *3TC + STV + RAL* | 1 | 0.27 | 17 | 4.51 |
| *1. PRE-CP* | *3TC + ddl* | 1 | 0.27 | 18 | 4.77 |
| *1. PRE-CP* | *3TC + ddl + DRV + RTV* | 1 | 0.27 | 19 | 5.04 |
| *1. PRE-CP* | *3TC/ABC + ATV* | 4 | 1.06 | 23 | 6.10 |
| *1. PRE-CP* | *3TC/ABC + ATV + LPV/r* | 1 | 0.27 | 24 | 6.37 |
| *1. PRE-CP* | *3TC/ABC + ATV + RTV* | 8 | 2.12 | 32 | 8.49 |
| *1. PRE-CP* | *3TC/ABC + DRV + RTV* | 2 | 0.53 | 34 | 9.02 |
| *1. PRE-CP* | *3TC/ABC + EFV* | 2 | 0.53 | 36 | 9.55 |
| *1. PRE-CP* | *3TC/ABC + LPV/r* | 1 | 0.27 | 37 | 9.81 |
| *1. PRE-CP* | *3TC/ABC + MVC* | 1 | 0.27 | 38 | 10.08 |
| *1. PRE-CP* | *3TC/ABC + RAL* | 1 | 0.27 | 39 | 10.34 |
| *1. PRE-CP* | *3TC/ABC + RTV* | 1 | 0.27 | 40 | 10.61 |
| *1. PRE-CP* | *3TC/ABC + TDF + ATV* | 1 | 0.27 | 41 | 10.88 |
| *1. PRE-CP* | *3TC/ABC/AZT + FPV + RTV* | 1 | 0.27 | 42 | 11.14 |
| *1. PRE-CP* | *3TC/AZT + ATV* | 2 | 0.53 | 44 | 11.67 |
| *1. PRE-CP* | *3TC/AZT + ATV + RTV* | 3 | 0.80 | 47 | 12.47 |
| *1. PRE-CP* | *3TC/AZT + DRV + RTV* | 2 | 0.53 | 49 | 13.00 |
| *1. PRE-CP* | *3TC/AZT + LPV/r* | 1 | 0.27 | 50 | 13.26 |
| *1. PRE-CP* | *ATV + MVC + RAL* | 1 | 0.27 | 51 | 13.53 |
| *1. PRE-CP* | *ATV + MVC + RAL + RTV* | 1 | 0.27 | 52 | 13.79 |
| *1. PRE-CP* | *ATV + MVC + RTV* | 1 | 0.27 | 53 | 14.06 |
| *1. PRE-CP* | *ATV + MVC + RTV + TDF* | 2 | 0.53 | 55 | 14.59 |
| *1. PRE-CP* | *ATV + MVC + RTV + TDF/FTC* | 1 | 0.27 | 56 | 14.85 |
| *1. PRE-CP* | *ATV + RAL* | 5 | 1.33 | 61 | 16.18 |
| *1. PRE-CP* | *ATV + RAL + RTV* | 1 | 0.27 | 62 | 16.45 |
| *1. PRE-CP* | *ATV + RTV* | 5 | 1.33 | 67 | 17.77 |
| *1. PRE-CP* | *ATV + RTV + ETV* | 1 | 0.27 | 68 | 18.04 |
| *1. PRE-CP* | *ATV + RTV + TDF/FTC* | 28 | 7.43 | 96 | 25.46 |
| *1. PRE-CP* | *ATV + TDF/FTC* | 3 | 0.80 | 99 | 26.26 |
| *1. PRE-CP* | *ATV + ddl + NVP + RTV* | 1 | 0.27 | 100 | 26.53 |
| *1. PRE-CP* | *DRV + EFV + FTC + RTV* | 1 | 0.27 | 101 | 26.79 |
| *1. PRE-CP* | *DRV + EFV + RAL + RTV* | 1 | 0.27 | 102 | 27.06 |
| *1. PRE-CP* | *DRV + ETV + MVC + RAL + RTV* | 1 | 0.27 | 103 | 27.32 |
| *1. PRE-CP* | *DRV + ETV + RAL + RTV* | 2 | 0.53 | 105 | 27.85 |
| *1. PRE-CP* | *DRV + ETV + RTV* | 1 | 0.27 | 106 | 28.12 |
| *1. PRE-CP* | *DRV + ETV + RTV + TDF* | 1 | 0.27 | 107 | 28.38 |
| *1. PRE-CP* | *DRV + MVC + RAL + RTV* | 4 | 1.06 | 111 | 29.44 |
| *1. PRE-CP* | *DRV + MVC + RTV* | 1 | 0.27 | 112 | 29.71 |
| *1. PRE-CP* | *DRV + MVC + RTV + TDF/FTC* | 2 | 0.53 | 114 | 30.24 |
| *1. PRE-CP* | *DRV + RAL + RTV* | 10 | 2.65 | 124 | 32.89 |
| *1. PRE-CP* | *DRV + RAL + RTV + TDF* | 2 | 0.53 | 126 | 33.42 |
| *1. PRE-CP* | *DRV + RAL + RTV + TDF/FTC* | 2 | 0.53 | 128 | 33.95 |
| *1. PRE-CP* | *DRV + RTV* | 5 | 1.33 | 133 | 35.28 |
| *1. PRE-CP* | *DRV + RTV + TDF + MVC* | 1 | 0.27 | 134 | 35.54 |
| *1. PRE-CP* | *DRV + RTV + TDF/FTC* | 23 | 6.10 | 157 | 41.64 |
| *1. PRE-CP* | *DRV + TDF/FTC* | 1 | 0.27 | 158 | 41.91 |
| *1. PRE-CP* | *EFV + TDF/FTC* | 3 | 0.80 | 161 | 42.71 |
| *1. PRE-CP* | *ETV + MVC + RAL* | 5 | 1.33 | 166 | 44.03 |
| *1. PRE-CP* | *ETV + MVC + TDF* | 1 | 0.27 | 167 | 44.30 |
| *1. PRE-CP* | *ETV + RAL* | 1 | 0.27 | 168 | 44.56 |
| *1. PRE-CP* | *FPV + MVC + RAL + RTV* | 1 | 0.27 | 169 | 44.83 |
| *1. PRE-CP* | *FPV + RTV + TDF/FTC* | 1 | 0.27 | 170 | 45.09 |
| *1. PRE-CP* | *FPV + TDF/FTC* | 2 | 0.53 | 172 | 45.62 |
| *1. PRE-CP* | *LPV/r* | 5 | 1.33 | 177 | 46.95 |
| *1. PRE-CP* | *LPV/r + RAL* | 4 | 1.06 | 181 | 48.01 |
| *1. PRE-CP* | *LPV/r + RAL + TDF/FTC* | 1 | 0.27 | 182 | 48.28 |
| *1. PRE-CP* | *LPV/r + TDF/FTC* | 12 | 3.18 | 194 | 51.46 |
| *1. PRE-CP* | *MVC + ETV* | 1 | 0.27 | 195 | 51.72 |
| *1. PRE-CP* | *MVC + TDF/FTC* | 2 | 0.53 | 197 | 52.25 |
| *1. PRE-CP* | *NVP + DRV + RTV* | 1 | 0.27 | 198 | 52.52 |
| *1. PRE-CP* | *NVP + TDF/FTC* | 3 | 0.80 | 201 | 53.32 |
| *1. PRE-CP* | *RAL + TDF/FTC* | 5 | 1.33 | 206 | 54.64 |
| *1. PRE-CP* | *TDF/FTC* | 4 | 1.06 | 210 | 55.70 |
| *1. PRE-CP* | *TDF/FTC/EFV* | 16 | 4.24 | 226 | 59.95 |
| *1. PRE-CP* | *sospensione* | 1 | 0.27 | 227 | 60.21 |
| *2. POST-CP* | *3TC* | 2 | 0.53 | 229 | 60.74 |
| *2. POST-CP* | *3TC + ATV* | 1 | 0.27 | 230 | 61.01 |
| *2. POST-CP* | *3TC + ATV + RTV* | 3 | 0.80 | 233 | 61.80 |
| *2. POST-CP* | *3TC + DRV + RTV* | 2 | 0.53 | 235 | 62.33 |
| *2. POST-CP* | *3TC + DRV + RTV + TDF* | 2 | 0.53 | 237 | 62.86 |
| *2. POST-CP* | *3TC + NVP + RAL* | 1 | 0.27 | 238 | 63.13 |
| *2. POST-CP* | *3TC + ddl + LPV/r* | 1 | 0.27 | 239 | 63.40 |
| *2. POST-CP* | *3TC/ABC + ATV* | 1 | 0.27 | 240 | 63.66 |
| *2. POST-CP* | *3TC/ABC + ATV + RTV* | 4 | 1.06 | 244 | 64.72 |
| *2. POST-CP* | *3TC/ABC + DRV + RTV* | 3 | 0.80 | 247 | 65.52 |
| *2. POST-CP* | *3TC/ABC + ETV* | 1 | 0.27 | 248 | 65.78 |
| *2. POST-CP* | *3TC/ABC + LPV/r* | 1 | 0.27 | 249 | 66.05 |
| *2. POST-CP* | *3TC/ABC + NVP* | 2 | 0.53 | 251 | 66.58 |
| *2. POST-CP* | *3TC/ABC + NVP+MVC* | 1 | 0.27 | 252 | 66.84 |
| *2. POST-CP* | *3TC/AZT + ATV + RTV* | 1 | 0.27 | 253 | 67.11 |
| *2. POST-CP* | *3TC/AZT + DRV + RTV* | 1 | 0.27 | 254 | 67.37 |
| *2. POST-CP* | *3TC/AZT + ETV* | 2 | 0.53 | 256 | 67.90 |
| *2. POST-CP* | *3TC/AZT + LPV/r* | 1 | 0.27 | 257 | 68.17 |
| *2. POST-CP* | *ATV + MVC + RTV* | 1 | 0.27 | 258 | 68.44 |
| *2. POST-CP* | *ATV + RTV* | 2 | 0.53 | 260 | 68.97 |
| *2. POST-CP* | *ATV + RTV + TDF/FTC* | 23 | 6.10 | 283 | 75.07 |
| *2. POST-CP* | *ATV + TDF/FTC* | 2 | 0.53 | 285 | 75.60 |
| *2. POST-CP* | *AZT + ATV + RAL* | 1 | 0.27 | 286 | 75.86 |
| *2. POST-CP* | *DRV + ETV + MVC + RTV* | 1 | 0.27 | 287 | 76.13 |
| *2. POST-CP* | *DRV + ETV + RAL + RTV* | 2 | 0.53 | 289 | 76.66 |
| *2. POST-CP* | *DRV + ETV + RAL + RTV + TDF/FTC* | 1 | 0.27 | 290 | 76.92 |
| *2. POST-CP* | *DRV + ETV + RTV* | 7 | 1.86 | 297 | 78.78 |
| *2. POST-CP* | *DRV + ETV + RTV + TDF* | 1 | 0.27 | 298 | 79.05 |
| *2. POST-CP* | *DRV + MVC + RAL + RTV* | 2 | 0.53 | 300 | 79.58 |
| *2. POST-CP* | *DRV + MVC + RTV* | 4 | 1.06 | 304 | 80.64 |
| *2. POST-CP* | *DRV + MVC + RTV + AZT* | 1 | 0.27 | 305 | 80.90 |
| *2. POST-CP* | *DRV + MVC + RTV + TDF/FTC* | 1 | 0.27 | 306 | 81.17 |
| *2. POST-CP* | *DRV + RAL + RTV* | 13 | 3.45 | 319 | 84.62 |
| *2. POST-CP* | *DRV + RAL + RTV + TDF* | 2 | 0.53 | 321 | 85.15 |
| *2. POST-CP* | *DRV + RAL + RTV + TDF/FTC* | 2 | 0.53 | 323 | 85.68 |
| *2. POST-CP* | *DRV + RTV* | 2 | 0.53 | 325 | 86.21 |
| *2. POST-CP* | *DRV + RTV + TDF* | 1 | 0.27 | 326 | 86.47 |
| *2. POST-CP* | *DRV + RTV + TDF + MVC* | 1 | 0.27 | 327 | 86.74 |
| *2. POST-CP* | *DRV + RTV + TDF/FTC* | 17 | 4.51 | 344 | 91.25 |
| *2. POST-CP* | *DRV + TDF/FTC* | 1 | 0.27 | 345 | 91.51 |
| *2. POST-CP* | *ETV + TDF/FTC* | 4 | 1.06 | 349 | 92.57 |
| *2. POST-CP* | *FPV + RTV + TDF/FTC* | 1 | 0.27 | 350 | 92.84 |
| *2. POST-CP* | *FPV + TDF/FTC* | 1 | 0.27 | 351 | 93.10 |
| *2. POST-CP* | *LPV/r + RAL* | 2 | 0.53 | 353 | 93.63 |
| *2. POST-CP* | *LPV/r + RAL + 3TC* | 1 | 0.27 | 354 | 93.90 |
| *2. POST-CP* | *LPV/r + TDF/FTC* | 9 | 2.39 | 363 | 96.29 |
| *2. POST-CP* | *MVC + DRV + RAL + RTV* | 1 | 0.27 | 364 | 96.55 |
| *2. POST-CP* | *MVC + RAL + TDF* | 1 | 0.27 | 365 | 96.82 |
| *2. POST-CP* | *NVP + TDF/FTC* | 2 | 0.53 | 367 | 97.35 |
| *2. POST-CP* | *RAL + TDF/FTC* | 5 | 1.33 | 372 | 98.67 |
| *2. POST-CP* | *TDF + ATV + RTV + ABC* | 1 | 0.27 | 373 | 98.94 |
| *2. POST-CP* | *TDF/FTC/EFV* | 4 | 1.06 | 377 | 100.00 |
